# Uncovering the Complexity Mechanism of Different Formulas Treatment for Rheumatoid Arthritis Based on a Novel Network Pharmacology Model

**DOI:** 10.3389/fphar.2020.01035

**Published:** 2020-07-10

**Authors:** Ke-xin Wang, Yao Gao, Cheng Lu, Yao Li, Bo-ya Zhou, Xue-mei Qin, Guan-hua Du, Li Gao, Dao-gang Guan, Ai-ping Lu

**Affiliations:** ^1^ Modern Research Center for Traditional Chinese Medicine, Shanxi University, Taiyuan, China; ^2^ Institute of Integrated Bioinformedicine and Translational Science, Hong Kong Baptist University, Hong Kong, Hong Kong; ^3^ Institute of Basic Research in Clinical Medicine, China Academy of Chinese Medical Sciences, Beijing, China; ^4^ Department of Ultrasound, Eighth Affiliated Hospital of Sun Yat-sen University, Guangzhou, China; ^5^ Institute of Materia Medica, Chinese Academy of Medical Sciences & Peking Union Medical College, Beijing, China; ^6^ Department of Biochemistry and Molecular Biology, School of Basic Medical Sciences, Southern Medical University, Guangzhou, China; ^7^ Guangdong Key Laboratory of Single Cell Technology and Application, Southern Medical University, Guangzhou, China

**Keywords:** Traditional Chinese medicine (TCM), rheumatoid arthritis, key gene network motif with significant (KNMS), mechanisms, network pharmacology

## Abstract

Traditional Chinese medicine (TCM) with the characteristics of “multi-component-multi-target-multi-pathway” has obvious advantages in the prevention and treatment of complex diseases, especially in the aspects of “treating the same disease with different treatments”. However, there are still some problems such as unclear substance basis and molecular mechanism of the effectiveness of formula. Network pharmacology is a new strategy based on system biology and poly-pharmacology, which could observe the intervention of drugs on disease networks at systematical and comprehensive level, and especially suitable for study of complex TCM systems. Rheumatoid arthritis (RA) is a chronic inflammatory autoimmune disease, causing articular and extra articular dysfunctions among patients, it could lead to irreversible joint damage or disability if left untreated. TCM formulas, Danggui-Sini-decoction (DSD), Guizhi-Fuzi-decoction (GFD), and Huangqi-Guizhi-Wuwu-Decoction (HGWD), et al., have been found successful in controlling RA in clinical applications. Here, a network pharmacology-based approach was established. With this model, key gene network motif with significant (KNMS) of three formulas were predicted, and the molecular mechanism of different formula in the treatment of rheumatoid arthritis (RA) was inferred based on these KNMSs. The results show that the KNMSs predicted by the model kept a high consistency with the corresponding C-T network in coverage of RA pathogenic genes, coverage of functional pathways and cumulative contribution of key nodes, which confirmed the reliability and accuracy of our proposed KNMS prediction strategy. All validated KNMSs of each RA therapy-related formula were employed to decode the mechanisms of different formulas treat the same disease. Finally, the key components in KNMSs of each formula were evaluated by *in vitro* experiments. Our proposed KNMS prediction and validation strategy provides methodological reference for interpreting the optimization of core components group and inference of molecular mechanism of formula in the treatment of complex diseases in TCM.

## Introduction

Rheumatoid Arthritis (RA) is a chronic systemic autoimmune disease with symmetric inflammation of aggressive multiple joints (Sodhi et al., 2015). As the most common inflammatory rheumatic disease, the prevalence of RA is about 0.5%-1.0% in the world (Saraux et al., 2006). The inflammatory cell infiltration of synovium, pannus formation, and the progressive destruction of articular cartilage and bone destruction are the main pathological properties of RA (Brzustewicz and Bryl, 2015). The data from epidemiological investigations shows that about 90% of RA patients developed bone erosions within 2 years, eventually leading to joint deformities or even disability (Cecilia et al., 2013). Therefore, RA brings great impact on the quality of life of patients and also imposes a heavy burden on families and society.

Traditional Chinese medicine (TCM) has the advantages of definite curative effect, safety and few side effects in the treatment of rheumatoid arthritis and has attracted more and more attention in the prevention and treatment of rheumatoid arthritis. TCM usually treats RA and other complex diseases in the form of formulas, which has theoretical advantages and rich clinical experience. In the study of RA therapy-related formulas, increasing evidence confirmed that different formulas can treat RA, which coincide with the theoretical concept of “treating the same disease with different treatments” in TCM (Fu et al., 2014). Such as Danggui-Sini-decoction (DSD) (Bang et al., 2017), Guizhi-Fuzi-decoction (GFD) (Peng et al., 2013), and Huangqi-Guizhi Wuwu-Decoction (HGWD) (Wang et al., 2010) etc., have been found successful in controlling RA in TCM clinics. Previous pharmacological studies have shown that DSD exert positive effects and good anti-inflammatory function which might protect collagen-induced arthritis rats from bone and cartilage destruction (Cheng et al., 2017). It has been reported that GFD could substantially inhibit the activities of interleukin-6 and tumor necrosis factor-α in the serum of adjuvant-induced arthritis rats, as well as inhibit the formation of synovitis and pannus, and has obvious therapeutic effect on rheumatoid arthritis (He and Gu, 2008; Xia and Song, 2011). In addition, some pharmacological experimental studies have found that HGWD could promote the apoptosis of synovial cells in rheumatoid arthritis rats with abnormal hyperfunction (Liu et al., 2017), and reduce the degree of foot swelling in adjuvant arthritis rats, affect the arthritis index of rats, and play a role in treating rheumatoid arthritis (Shi et al., 2006).

In these formulas, DSD consists of 7 herbs: *Angelica sinensis* (Oliv.) Diels (Danggui, 12 g), *Cinnamomum cassia* (L.) J. Presl (Cinnamomi ramulus, Guizhi, 9 g), *Paeonia lactiflora* Pall. (Baishao, 9 g), *Asarum sieboldii* Miq. (Xixin, 3 g), *Glycyrrhiza uralensis* Fisch. ex DC. (Gancao, 6 g), *Tetrapanax papyrifer* (Hook.) K. Koch (Medulla tetrapanacis, Tongcao, 6 g), *Ziziphus jujuba* Mill. (Jujubae fructus, Dazao, 8). GFD consists of 5 herbs: *Cinnamomum cassia* (L.) J. Presl (Cinnamomi ramulus, Guizhi, 12 g), *Aconitum carmichaeli* Debeaux (Aconiti lateralis radix praeparata, Fuzi, 15 g), *Zingiber officinale* Roscoe (Shengjiang, 9 g), *Glycyrrhiza uralensis* Fisch. ex DC. (Gancao, 6 g), *Ziziphus jujuba* Mill. (Jujubae fructus, Dazao, 12). HGWD consists of 5 herbs: *Astragalus mongholicus* Bunge (Huangqi, 15 g), *Paeonia lactiflora* Pall. (Baishao, 12 g), *Cinnamomum cassia* (L.) J. Presl (Cinnamomi ramulus, Gui zhi, 12 g), *Zingiber officinale* Roscoe (Shengjiang, 25 g), *Ziziphus jujuba* Mill. (Jujubae fructus, Dazao, 4). These traditional formulas are recorded in the Chinese pharmacopoeia (National Pharmacopoeia Commission, 2015). However, the molecular mechanism of these different formulas in treating rheumatoid arthritis under the concept of “treating the same disease with different treatments” is still unclear. How to develop new methods to detect the key component groups of different formulas for treating rheumatoid arthritis and speculate the possible mechanism not only provides the benefit therapy strategy for the precise treatment of RA, but also provides methodological reference for the analysis of the mechanism of treating the same disease with different treatments in TCM.

Network pharmacology has been widely used in the research of treating the same diseases with different formulas. For example, Gao et al. used network pharmacology to decode the mechanisms of Xiaoyao powder and Kaixin powder in treating depression; Liu et al. clarified the molecular mechanism of Sini San and Suanzaoren Tang in treating insomnia based on network pharmacology, etc (Yao et al., 2018; Liu et al., 2019). With the in-depth intersection of systems biology, poly-pharmacology, bioinformatics and other technologies, and the continuous improvement of the accuracy, reliability, and integrity of data resources, the research ideas and technical means of network pharmacology will be better applied to the mechanism research of formulas in TCM and provide more innovation in methodology for the molecular level research of TCM.

In this study, network pharmacology model was applied to analyze the key gene network motif with significant (KNMS) of different formulas in the treatment of RA. Coverage of RA pathogenic genes, coverage of functional pathways and cumulative contribution of key nodes were employed to evaluate the accuracy and reliability of KNMSs, and then the validated KNMSs were used to infer the common potential mechanism of different formulas in the treatment of RA. In summary, the proposed network pharmacology strategy aims to identify major mechanism and related pharmacological effects of different treatments in treating RA through specific KNMSs, which may offer a new network-based method for evaluating and selecting suitable treatment strategies of complex diseases in TCM.

## Materials and Methods

### Flowchart

**Figure 1:**
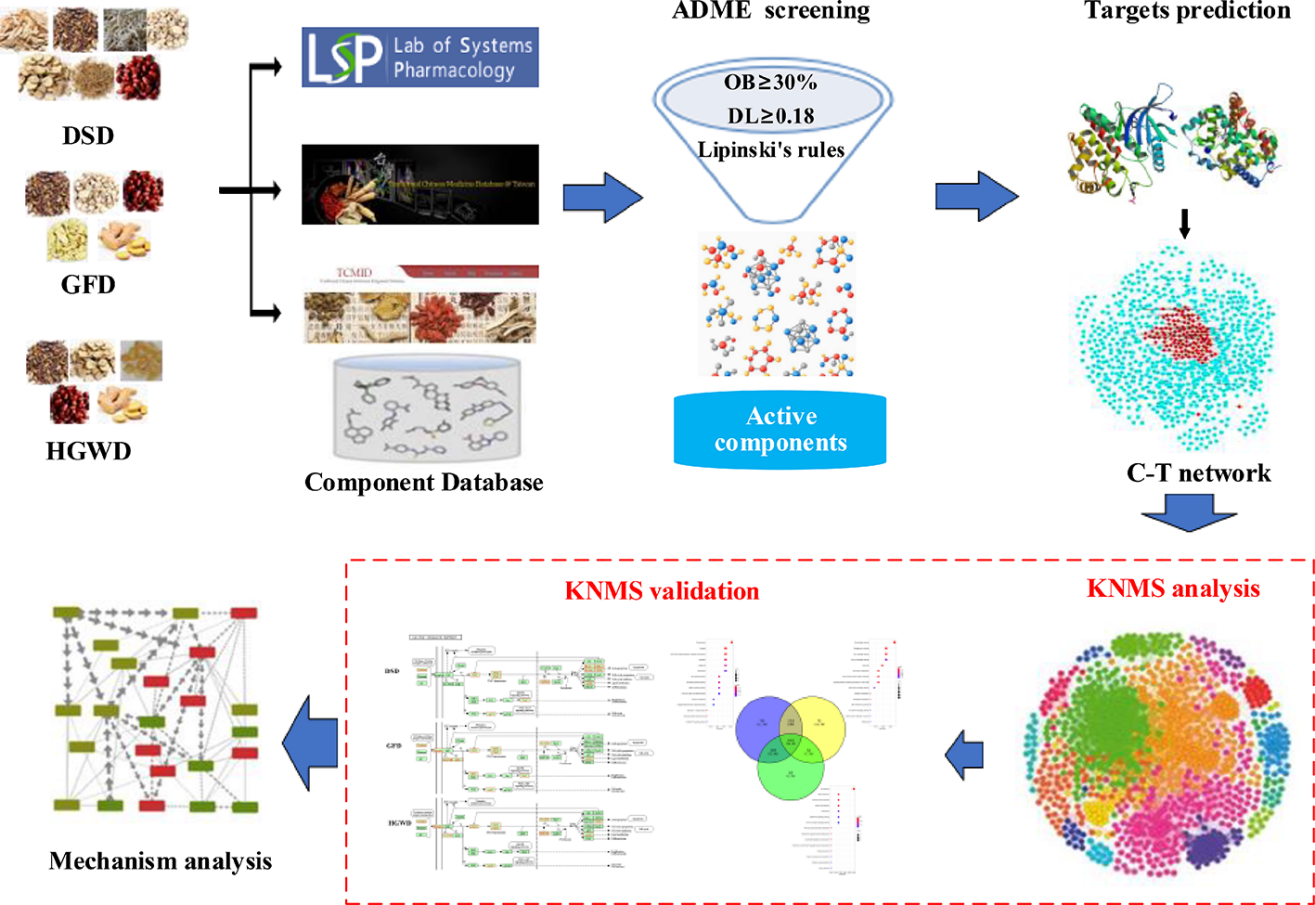
A ****schematic diagram of network pharmacology-based strategy to decode the mechanisms of different formulas treat the same disease of TCM. DSD represents Danggui-Sini-decoction, GFD represents Guizhi-Fuzi-decoction, and HGWD represents Huangqi-Guizhi Wuwu-Decoction, KNMSs represents key gene network motifs with significant.

This phenomenon that different formulas treat the same diseases is widely used in TCM clinical applications. However, there is lack of systematic method to decode the mechanisms of treat the same disease with different treatments. In this study, we designed a network pharmacology model to decode the common and specific potential mechanisms of 3 formulas in the treatment of RA, which may provide a methodological reference for different formulas treat the same disease. The workflow is illustrated in **Figure 1** and described as follows: 1) the components of DSD, GFD and HGWD were collected from TCMSP, TCMID, and TCM@Taiwan; 2) ADME based methods were used to identify the main active components; 3) the main active components from three formulas and their predicted targets were used to construct the component-target (C-T) networks; 4) The KNMSs were detected from integrated C-T and target-target interaction networks; 5) the KNMSs were validated by the coverage of RA pathogenic genes, coverage of functional pathways and cumulative contribution of key nodes; 6) Finally, all validated KNMSs were employed to decode the underlying mechanism of different formulas treat the same disease.

### Component Identification

All chemical components of Danggui-Sini-decoction (DSD), Guizhi-Fuzi-decoction (GFD), and Huangqi-Guizhi Wuwu-Decoction (HGWD) were collected from Traditional Chinese Medicine Systems Pharmacology (TCMSP) Database (Ru et al., 2014) (http://lsp.nwsuaf.edu.cn/tcmsp.php), Traditional Chinese Medicine integrated database (Xue et al., 2013) (TCMID, http://www.megabionet.org/tcmid/), and TCM@Taiwan (Chen, 2012) (http://tcm.cmu.edu.tw/zh-tw). The chemical identification and concentration of the herbs in DSD, GFD, and HGWD were collected from the previous reports. All chemical structures were prepared and converted into canonical SMILES using Open Babel Toolkit (version 2.4.1). The targets of DSD, GFD, and HGWD were predicted by using Similarity Ensemble Approach SEA (Keiser et al., 2007) (http://sea.bkslab.org/) and Swiss Target Prediction (David et al., 2014) (http://www.swisstargetprediction.ch/).

### ADME Screening

Components that meet the Lipinski’ rules of five usually have better pharmacokinetic properties, higher bioavailability during metabolism in the body, and are therefore more likely to be drug candidates (Lipinski et al., 2012; Damião et al., 2014). Oral bioavailability (OB) refers to the extent and rate of active components release from the herbs into the systemic circulation and is an important indicator for evaluating the intrinsic quality of the component (Xu et al., 2012). Drug-like (DL) indicate the characteristics that an ideal drug should have and was a comprehensive reflection of the physical and chemical properties and structural characteristics exhibited by successful drugs (Tao et al., 2013). In this study, active components from DSD, GFD, and HGWD were mainly filtered by integrating Lipinski’s rules, oral bioavailability (OB), and drug-likeness (DL). The detail of Lipinski’s rules includes molecular weight lower than 500 Da, number of donor hydrogen bonds less than 5, number of acceptor hydrogen bonds less than 10, the log*P* lower than 5 and over -2, and meets only the criteria of 10 or fewer rotatable bonds. Besides, oral bioavailability (OB), and drug-likeness (DL) also were employed to screen the active components. The components with OB values higher than 30% and DL values higher than 0.14 were retained for further investigation (Wang et al., 2018).

### Networks Construction

The component-target (C-T) networks of three formulas were constructed by using Cytoscape software (Version 3.7.0) (Lopes et al., 2010). The topological parameters of networks were analyzed using Cytoscape plugin NetworkAnalyzer (Jong et al., 2003).

### Detection of Key Gene Network Motif With Significant (KNMS)

The exploration of motif structures in networks is an important issue in many domains and disciplines. To find key gene network motifs with significant (KNMS) of 3 formulas in the treatment of RA, a mathematical algorithm was designed and described as follows:

To take advantage of the motif structure of the network, *m motif codebooks,* and one index codebook are used to describe the random walker’s movements within and between motifs, respectively. Motif codebook *i* has one codeword for each node *α∈i* and one exit codeword. The codeword lengths are derived from the frequencies at which the random walker visits each of the nodes in the motif, *p_α__∈__i_*, and exits the motif, *q*_*i*↷_. We use *p*_*i*↻_ to denote the sum of these frequencies, the total use of codewords in motif *i*, and *P^i^* to denote the normalized probability distribution. Similarly, the index codebook has codewords for motif entries. The codeword lengths are derived from the set of frequencies at which the random walker enters each motif, *q*_*i*↶_. We use *q*_↶_ to denote the sum of these frequencies, the total use of codewords to move into motifs, and *Q* to denote the normalized probability distribution. We want to express average length of codewords from the index codebook and the motif codebooks weighted by their rates of use. Therefore, the map equation is

$$L\left(\text{M}\right)=q_{↶}H\left(Q\right)+\sum_{i=1}^{m}p_{i↻}H\left(p_{i}\right)$$

Below we explain the terms of the map equation in detail and we provide examples with Huffman codes for illustration.

*L*(M) represents the per-step description length for motif partition *M*. That is, for motif partition *M* of n nodes into *m* motifs, the lower bound of the average length of the code describing a step of the random walker.

$$q_{↶}=\sum_{i=1}^{m}q_{i↶}$$

The rate at which the index codebook is used. The per-step use rate of the index codebook is given by the total probability that the random walker enters any of them motifs. This variable represents the proportion of all codes representing motif names in the codes. Where *q*_*i*↶_ is probability of jumping out of Motif *i*.

$$\;\;\;H\left(Q\right)=-\sum_{i=1}^{m}\left(q_{i↶}/q_{i↶})\text{log}(q_{i↶}/q_{↶}\right)$$

This variable represents the average byte length required to encode motif names. The frequency-weighted average length of codewords in the index codebook. The entropy of the relative rates to use the motif codebooks measures the smallest average codeword length that is theoretically possible. The heights of individual blocks under *Index codebook* correspond to the relative rates and the codeword lengths approximately correspond to the negative logarithm of the rates in base 2.

$$p_{i↻}=\sum_{\alpha \in i}p_{\alpha }+q_{i↷}$$

This variable represents the coding proportion of all nodes (including jump-out nodes) belonging to motif *i* in the coding. The rate at which the motif codebook *i* is used, which is given by the total probability that any node in the motif is visited, plus the probability that the random walker exits the motif and the exit codeword is used.

$$H\left(p^{i}\right)=-(q_{i↷}/p_{i↻})\text{log}(q_{i↷}/p_{i↻})-\sum_{\alpha \in i}\left(p_{a}/p_{i↻})\text{log}(p_{a}/p_{i↻}\right)$$

This variable represents the average byte length required to encode all nodes in motif *i*. The frequency-weighted average length of codewords in motif codebook *i*. The entropy of the relative rates at which the random walker exits motif *i* and visits each node in motif *i* measures the smallest average codeword length that is theoretically possible. The heights of individual blocks under motif codebooks correspond to the relative rates and the codeword lengths approximately correspond to the negative logarithm of the rates in base 2.

### Contribution Coefficient Calculation

The contribution coefficient (CC) represents the network contribution of KNMSs in 3 formulas. R value was used to determine the importance of the components by the following mathematical model:

$$R=\frac{d_{c}-d_{c}(\text{min})}{d_{c}\max-d_{c}(\min)}$$

$$CC\left(i\right)=\frac{\Sigma_{i}^{n}R_{i}}{\Sigma_{i}^{n}R_{j}}\times 100\%$$

where *d_c_* represents the degree of each component, which is calculated by Cytoscape. *R* is an indicator to evaluate the importance of the component.

Where n is the number of components from different KNMSs of DSD, GFD, and HGWD, respectively; m is the number of components from C-T network of DSD, GFD, and HGWD, respectively; *Ri* represents the indicator of each component in KNMSs of DSD, GFD, and HGWD, and *Rj* represents the indicator of each component in C-T network of DSD, GFD, and HGWD.

### KEGG Pathway

To analyze the main function of the KNMSs, the pathway data were obtained from the Kyoto Encyclopedia of Genes and Genomes (KEGG) database (Draghici et al., 2007) for KEGG pathway enrichment analyses. P-values were set at 0.05 as the cut-off criterion. The results of analysis were annotated by Pathview (Luo and Brouwer, 2013) in the R Bioconductor package (https://www.bioconductor.org/).

### Experimental Validation

#### Materials

Isoliquiritigenin, isorhamnetin and quercetin (≥98% purity by HPLC) was obtained from Chengdu Pufei De Biotech Co., Ltd (Chengdu, China). Fetal bovine serum (FBS) and Dulbecco’s modified Eagle’s medium (DMEM) were purchased from Gibco (Grand Island, USA). Lipopolysaccharide (LPS) was purchased from Sigma-Aldrich Co., Ltd (St Louis, USA).

#### Cell Culture and Treatment

RAW264.7 cells were obtained from the cell bank of the Chinese Academy of Sciences (Shanghai, China). The cells were cultured in DMEM with 10% FBS, and incubated at 37°C under 5% CO_2_. When RAW264.7 cells reached 80% confluency, the cells were treated with isoliquiritigenin, isorhamnetin and quercetin for 2 h, then the cells were treated with LPS (1 μg/ml) for 24 h.

#### Cell Viability Assay

MTT assay was utilized to measure cell viability. RAW264.7 cells (6×10^4^ per/well) were seeded in 96-well plates. After 24 h incubation, RAW264.7 cells were treated with 1, 5, 10, 20, 40, and 80 μM isoliquiritigenin, isorhamnetin and quercetin for 24 h. Ten μl of MTT were added to reach a final concentration of 0.5 mg/ml, and incubated for a further 4 h. The absorbance was measured at 570 nm with a microplate reader (BioTek, USA).

#### Measurement of NO

Griess reagent was utilized to detected the level of NO in the culture supernatant of RAW264.7 cells. After incubation with isoliquiritigenin, isorhamnetin and quercetin for 2 h and LPS (1 μg/ml) for 24 h, the culture supernatant was collected and mixed with Griess reagent for NO assay. The absorbance was measured at 540 nm using a microplate reader.

### Statistical Analysis

To compare the importance of motifs in three formulas, SPSS22.0 was used for statistical analysis. One-way analysis of variance followed by a Dunnett post-hoc test was used to compare more than two groups. Obtained p-values were corrected by Benjamini-Hochberg false discovery rate (FDR). Results were considered as statistically significant if the p-value was <0.05.

## Results

### Chemical Analysis

Chemical analysis plays important roles in the study of substances basis and mechanism of herbs in the formulas. By searching from the literature, we collected the information on specific chemical identification and concentration of the herbs in DSD, GFD and HGWD, respectively. The detail information was shown in **Table 1** and **Table S1**. The results suggest that chemical components of herbs and the concentration of identified components provide an experiment-aided chemical space for search of active components. This will provide valuable reference for the further analysis.

**Table 1:** The information on chemical analysis of the herbs from the literature in DSD, GFD, and HGWD.

Herb	Method	Component	Concentration	Formula	Ref.
*Angelica sinensis* (Oliv.) Diels (Danggui)	HPLC	Ferulic acid	0.36 mg/g	DSD	Xie et al., 2007
		Coniferylferulate	6.11 mg/g		
		*Z*-ligustilide	4.34 mg/g		
		*E*-ligustilide	0.23 mg/g		
		*Z*-3-butylidenephthalide	0.20 mg/g		
		*E*-3-butylidenephthalide	0.08 mg/g		
*Cinnamomum cassia* (L.) J. Presl (Cinnamomi ramulus, Guizhi)	UHPLC	Protocatechuic acid	0.11 mg/g	DSD, GFD, HGWD	Liang et al., 2011
		Coumarin	0.84 mg/g		
		Cinnamic alcohol	0.04 mg/g		
		Cinnamic acid	0.68 mg/g		
		Cinnamaldehyde	9.93 mg/g		
*Paeonia lactiflora* Pall. (Baishao)	HPLC	Gallic acid	2.33 mg/g	DSD, HGWD	Li et al., 2011
		Hydroxyl-paeoniflorin	1.89 mg/g		
		Catechin	0.03 mg/g		
		Albiflorin	4.44 mg/g		
		Paeoniflorin	4.81 mg/g		
		Benzoic acid	0.03 mg/g		
		1, 2, 3, 4, 6 -pentagalloylglucose	4.80 mg/g		
		Benzoyl -paeoniflorin	0.11 mg/g		
		Paeonol	0.07 mg/g		
*Asarum sieboldii* Miq. (Xixin)	HPLC	Aristolochic acid A	0.009 mg/g	DSD	Gao et al., 2005
*Glycyrrhiza uralensis* Fisch. ex DC. (Gancao)	HPLC	Glycyrrhizin	97.49 mg/g	DSD, GFD	Chen et al., 2009
		Liquiritin	102.83 mg/g		
		Lsoliquritigenin	98.30 mg/g		
*Tetrapanax papyrifer* (Hook.) K.Koch (Medulla tetrapanacis, Tongcao)	RP- HPLC	Calceolar ioside B	0.86 mg/g	DSD	Gao et al., 2007
*Ziziphus jujuba* Mill. (Jujubae fructus (Dazao)	HPLC	Rutin	0.21 mg/g	DSD, GFD, HGWD	Wang et al., 2013
		Quercetin	0.008 mg/g		
		Isorhamnetin	0.17 mg/g		
*Aconitum carmichaeli* Debeaux (Aconiti lateralis radix praeparata, Fuzi)	HPLC	aconitine	0.28 mg/g	GFD	Sun et al., 2009
		hypaconitine	0.70 mg/g		
		mesaconitine	1.04 mg/g		
		benzoylaconine	0.009 mg/g		
		benzoylhypaconine	0.007 mg/g		
		benzoylmesaconin	0.07 mg/g		
*Zingiber officinale* Roscoe (Shengjiang)	HPLC	6-Gingerol	16.62 mg/g	GFD, HGWD	Zhang et al., 2009
		6-Shogaol	4.92 mg/g		
*Astragalus mongholicus* Bunge (Huangqi)	HPLC	Campanulin	0.42 mg/g	HGWD	Li et al., 2015
		Formononetin	0.02 mg/g		

### Active Components in DSD, GFD, and HGWD

**Figure 2:**
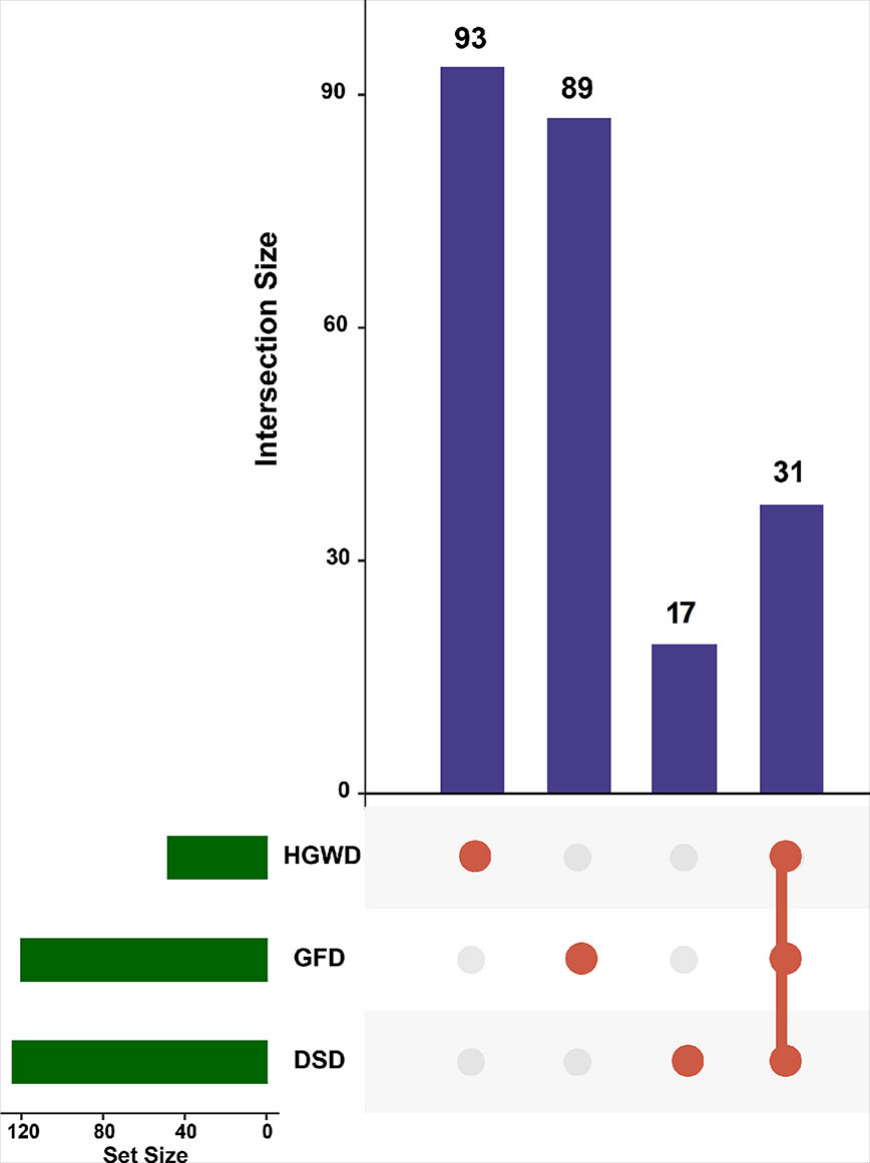
Distribution map of active components in DSD, GFD, and HGWD.

By a comprehensive search of the TCMSP, TCMID, and TCM@Taiwan database, 812 components from seven herbs in DSD, 640 components from five herbs in GFD, and 459 components from five herbs in HGWD were obtained. A TCM formula usually contains large number of components, and ADME screening approaches are always used to select active components. After ADME screening, 124 active components in DSD, 120 active components in GFD, and 48 active components in HGWD were passed the combined filtering criteria which integrated by Lipinski’s rule, OB, and DL (**Table 2**). For further analysis of these active components, 31 common components in three formulas and 93, 89, and 17 unique components in DSD, GFD, and HGWD were found (**Figure 2**). These results indicate that three formulas might exert roles in treating RA by affecting the common components and specific components.

**Table 2:** Components in DSD, GFD, and HGWD for further analysis after ADME screening.

ID	Component	MW	Logp	HDON	HACC	RBN	OB	DL	Source
DSD1	(+)-catechin	290.29	1.02	5	6	1	54.83	0.24	Baishao
DSD2	(3S,5R,8R,9R,10S,14S)-3,17-dihydroxy-4,4,8,10,14-pentamethyl-2,3,5,6,7,9-hexahydro-1H-cyclopenta [a]phenanthrene-15,16-dione	358.52	3.52	2	4	0	43.56	0.53	Baishao
DSD3	11alpha,12alpha-epoxy-3beta-23-dihydroxy-30-norolean-20-en-28,12beta-olide	470.71	3.82	2	5	1	64.77	0.38	Baishao
DSD4	albiflorin_qt	318.35	0.53	2	6	4	66.64	0.33	Baishao
DSD5	kaempferol	286.25	1.23	4	6	1	41.88	0.24	Baishao
DSD6	Lactiflorin	462.49	0.31	3	10	5	49.12	0.8	Baishao
DSD7	paeoniflorgenone	318.35	0.86	1	6	4	87.59	0.37	Baishao
DSD8	paeoniflorin_qt	318.35	0.69	2	6	4	68.18	0.4	Baishao
DSD9	(-)-catechin	290.29	1.02	5	6	1	49.68	0.24	Dazao
DSD10	(S)-Coclaurine	285.37	2.2	3	4	3	42.35	0.24	Dazao
DSD11	21302-79-4	486.76	4.53	3	5	3	73.52	0.77	Dazao
DSD12	berberine	336.39	3.75	0	4	2	36.86	0.78	Dazao
DSD13	coumestrol	268.23	2.43	2	5	0	32.49	0.34	Dazao
DSD14	Fumarine	353.4	1.95	0	6	0	59.26	0.83	Dazao
DSD15	Jujubasaponin V_qt	472.78	4.61	2	4	2	36.99	0.63	Dazao
DSD16	jujuboside A_qt	472.78	3.8	2	4	1	36.67	0.62	Dazao
DSD17	Jujuboside C_qt	472.78	3.8	2	4	1	40.26	0.62	Dazao
DSD18	malkangunin	432.56	2.72	2	7	6	57.71	0.63	Dazao
DSD19	Mauritine D	342.46	1.11	2	6	2	89.13	0.45	Dazao
DSD20	Moupinamide	313.38	2.46	3	5	6	86.71	0.26	Dazao
DSD21	Nuciferin	295.41	3.38	0	3	2	34.43	0.4	Dazao
DSD22	quercetin	302.25	1.07	5	7	1	46.43	0.28	Dazao
DSD23	Ruvoside_qt	390.57	1.42	3	5	2	36.12	0.76	Dazao
DSD24	Spiradine A	311.46	1.29	1	3	0	113.52	0.61	Dazao
DSD25	stepharine	297.38	1.76	1	4	2	31.55	0.33	Dazao
DSD26	Stepholidine	327.41	2.26	2	5	2	33.11	0.54	Dazao
DSD27	Ziziphin_qt	472.78	3.8	2	4	1	66.95	0.62	Dazao
DSD28	zizyphus saponin I_qt	472.78	3.8	2	4	1	32.69	0.62	Dazao
DSD29	2,6-di(phenyl)thiopyran-4-thione	280.43	4.39	0	0	2	69.13	0.15	Danggui
DSD30	(-)-Medicocarpin	432.46	1.26	4	9	4	40.99	0.95	Gancao
DSD31	(2R)-7-hydroxy-2-(4-hydroxyphenyl)chroman-4-one	256.27	2.79	2	4	1	71.12	0.18	Gancao
DSD32	(2S)-6-(2,4-dihydroxyphenyl)-2-(2-hydroxypropan-2-yl)-4-methoxy-2,3-dihydrofuro[3,2-g]chromen-7-one	384.41	2.61	3	7	3	60.25	0.63	Gancao
DSD33	(2S)-7-hydroxy-2-(4-hydroxyphenyl)-8-(3-methylbut-2-enyl)chroman-4-one	324.4	3.62	2	4	3	36.57	0.32	Gancao
DSD34	(E)-1-(2,4-dihydroxyphenyl)-3-(2,2-dimethylchromen-6-yl)prop-2-en-1-one	322.38	4.46	2	4	3	39.62	0.35	Gancao
DSD35	(E)-3-[3,4-dihydroxy-5-(3-methylbut-2-enyl)phenyl]-1-(2,4-dihydroxyphenyl)prop-2-en-1-one	340.4	3.47	4	5	5	46.27	0.31	Gancao
DSD36	1,3-dihydroxy-8,9-dimethoxy-6-benzofurano[3,2-c]chromenone	328.29	2.74	2	7	2	62.9	0.53	Gancao
DSD37	1,3-dihydroxy-9-methoxy-6-benzofurano[3,2-c]chromenone	298.26	2.48	2	6	1	48.14	0.43	Gancao
DSD38	18α-hydroxyglycyrrhetic acid	486.76	4.54	3	5	1	41.16	0.71	Gancao
DSD39	1-Methoxyphaseollidin	354.43	3.66	2	5	3	69.98	0.64	Gancao
DSD40	2-(3,4-dihydroxyphenyl)-5,7-dihydroxy-6-(3-methylbut-2-enyl)chromone	354.38	2.99	4	6	3	44.15	0.41	Gancao
DSD41	2-[(3R)-8,8-dimethyl-3,4-dihydro-2H-pyrano[6,5-f]chromen-3-yl]-5-methoxyphenol	338.43	4.35	1	4	2	36.21	0.52	Gancao
DSD42	3-(2,4-dihydroxyphenyl)-8-(1,1-dimethylprop-2-enyl)-7-hydroxy-5-methoxy-coumarin	368.41	3.92	3	6	4	59.62	0.43	Gancao
DSD43	3-(3,4-dihydroxyphenyl)-5,7-dihydroxy-8-(3-methylbut-2-enyl)chromone	354.38	3.02	4	6	3	66.37	0.41	Gancao
DSD44	3’-Hydroxy-4’-O-Methylglabridin	354.43	3.76	2	5	2	43.71	0.57	Gancao
DSD45	3’-Methoxyglabridin	354.43	3.76	2	5	2	46.16	0.57	Gancao
DSD46	5,7-dihydroxy-3-(4-methoxyphenyl)-8-(3-methylbut-2-enyl)chromone	352.41	3.29	2	5	4	30.49	0.41	Gancao
DSD47	6-prenylated eriodictyol	356.4	2.99	4	6	3	39.22	0.41	Gancao
DSD48	7,2’,4’-trihydroxy-5-methoxy-3-arylcoumarin	300.28	2.51	3	6	2	83.71	0.27	Gancao
DSD49	7-Acetoxy-2-methylisoflavone	294.32	3.41	0	4	3	38.92	0.26	Gancao
DSD50	7-Methoxy-2-methyl isoflavone	266.31	3.48	0	3	2	42.56	0.2	Gancao
DSD51	8-(6-hydroxy-2-benzofuranyl)-2,2-dimethyl-5-chromenol	308.35	4.27	2	4	1	58.44	0.38	Gancao
DSD52	8-prenylated eriodictyol	356.4	2.99	4	6	3	53.79	0.4	Gancao
DSD53	Calycosin	284.28	2.82	2	5	2	47.75	0.24	Gancao
DSD54	dehydroglyasperins C	340.4	3.11	4	5	3	53.82	0.37	Gancao
DSD55	DFV	256.27	2.79	2	4	1	32.76	0.18	Gancao
DSD56	echinatin	270.3	3.41	2	4	4	66.58	0.17	Gancao
DSD57	Eurycarpin A	338.38	3.29	3	5	3	43.28	0.37	Gancao
DSD58	formononetin	268.28	3.01	1	4	2	69.67	0.21	Gancao
DSD59	Gancaonin A	352.41	3.34	2	5	4	51.08	0.4	Gancao
DSD60	Gancaonin B	368.41	3.14	3	6	4	48.79	0.45	Gancao
DSD61	Gancaonin G	352.41	3.25	2	5	4	60.44	0.39	Gancao
DSD62	Gancaonin H	420.49	3.99	3	6	3	50.1	0.78	Gancao
DSD63	Glabranin	324.4	3.59	2	4	3	52.9	0.31	Gancao
DSD64	Glabrene	322.38	3.68	2	4	1	46.27	0.44	Gancao
DSD65	Glabridin	324.4	3.81	2	4	1	53.25	0.47	Gancao
DSD66	Glabrone	336.36	3.78	2	5	1	52.51	0.5	Gancao
DSD67	Glepidotin A	338.38	2.88	3	5	3	44.72	0.35	Gancao
DSD68	Glepidotin B	340.4	2.88	3	5	3	64.46	0.34	Gancao
DSD69	glyasperin B	370.43	3.14	3	6	4	65.22	0.44	Gancao
DSD70	Glyasperin C	356.45	3.53	3	5	4	45.56	0.4	Gancao
DSD71	glyasperin F	354.38	3.52	3	6	1	75.84	0.54	Gancao
DSD72	Glyasperins M	368.41	3.57	2	6	2	72.67	0.59	Gancao
DSD73	Glycyrin	382.44	3.78	2	6	5	52.61	0.47	Gancao
DSD74	Glycyrol	366.39	4.06	2	6	3	90.78	0.67	Gancao
DSD75	Glycyrrhiza flavonol A	370.38	2.18	4	7	1	41.28	0.6	Gancao
DSD76	Glypallichalcone	284.33	3.8	1	4	5	61.6	0.19	Gancao
DSD77	Glyzaglabrin	298.26	2.32	2	6	1	61.07	0.35	Gancao
DSD78	HMO	268.28	2.92	1	4	2	38.37	0.21	Gancao
DSD79	Inermine	284.28	2.19	1	5	0	75.18	0.54	Gancao
DSD80	Inflacoumarin A	322.38	4.36	2	4	3	39.71	0.33	Gancao
DSD81	Isoglycyrol	366.39	4.15	1	6	1	44.7	0.84	Gancao
DSD82	Isolicoflavonol	354.38	2.92	4	6	3	45.17	0.42	Gancao
DSD83	isoliquiritigenin	256.27	3.04	3	4	3	85.32	0.15	Gancao
DSD84	isorhamnetin	316.28	1.31	4	7	2	49.6	0.31	Gancao
DSD85	Isotrifoliol	298.26	2.54	2	6	1	31.94	0.42	Gancao
DSD86	Jaranol	314.31	2.8	2	6	3	50.83	0.29	Gancao
DSD87	kanzonols W	336.36	3.97	2	5	1	50.48	0.52	Gancao
DSD88	Licoagrocarpin	338.43	3.94	1	4	3	58.81	0.58	Gancao
DSD89	Licoagroisoflavone	336.36	2.95	2	5	2	57.28	0.49	Gancao
DSD90	licochalcone a	338.43	4.74	2	4	6	40.79	0.29	Gancao
DSD91	Licochalcone B	286.3	3.17	3	5	4	76.76	0.19	Gancao
DSD92	licochalcone G	354.43	4.21	3	5	6	49.25	0.32	Gancao
DSD93	Licocoumarone	340.4	4.1	3	5	4	33.21	0.36	Gancao
DSD94	licoisoflavanone	354.38	3.54	3	6	1	52.47	0.54	Gancao
DSD95	Licoisoflavone	354.38	2.99	4	6	3	41.61	0.42	Gancao
DSD96	Licoisoflavone B	352.36	3.54	3	6	1	38.93	0.55	Gancao
DSD97	licopyranocoumarin	384.41	2.47	3	7	3	80.36	0.65	Gancao
DSD98	Licoricone	382.44	3.08	2	6	5	63.58	0.47	Gancao
DSD99	liquiritin	418.43	0.43	5	9	4	65.69	0.74	Gancao
DSD100	Lupiwighteone	338.38	3.23	3	5	3	51.64	0.37	Gancao
DSD101	Medicarpin	270.3	3.07	1	4	1	49.22	0.34	Gancao
DSD102	naringenin	272.27	2.47	3	5	1	59.29	0.21	Gancao
DSD103	Odoratin	314.31	2.81	2	6	3	49.95	0.3	Gancao
DSD104	Phaseol	336.36	4.59	2	5	2	78.77	0.58	Gancao
DSD105	Phaseolinisoflavan	324.4	3.77	2	4	1	32.01	0.45	Gancao
DSD106	Pinocembrin	256.27	2.85	2	4	1	64.72	0.18	Gancao
DSD107	Quercetin der.	330.31	2.55	3	7	3	46.45	0.33	Gancao
DSD108	Semilicoisoflavone B	352.36	3.55	3	6	1	48.78	0.55	Gancao
DSD109	shinpterocarpin	322.38	4.13	1	4	0	80.3	0.73	Gancao
DSD110	Sigmoidin-B	356.4	3.02	4	6	3	34.88	0.41	Gancao
DSD111	Vestitol	272.32	2.89	2	4	2	74.66	0.21	Gancao
DSD112	ent-Epicatechin	290.29	2.83	5	6	1	48.96	0.24	Guizhi
DSD113	beta-sitosterol	414.79	3.2	1	1	6	36.91	0.75	Guizhi
DSD114	sitosterol	414.79	2.71	1	1	6	36.91	0.75	Guizhi
DSD115	(-)-taxifolin	304.27	1.66	5	7	1	60.51	0.27	Guizhi
DSD116	DMEP	282.32	1.93	0	6	10	55.66	0.15	Guizhi
DSD117	paryriogenin A	466.72	4.62	1	4	1	41.41	0.76	Tongcao
DSD118	(3S)-7-hydroxy-3-(2,3,4-trimethoxyphenyl)chroman-4-one	330.36	1.59	1	6	4	48.23	0.33	Xixin
DSD119	[(1S)-3-[(E)-but-2-enyl]-2-methyl-4-oxo-1-cyclopent-2-enyl] (1R,3R)-3-[(E)-3-methoxy-2-methyl-3-oxoprop-1-enyl]-2,2-dimethylcyclopropane-1-carboxylate	360.49	4.18	0	5	8	62.52	0.31	Xixin
DSD120	4,9-dimethoxy-1-vinyl-$b-carboline	254.31	2.96	0	3	3	65.3	0.19	Xixin
DSD121	Caribine	326.43	0.53	2	5	0	37.06	0.83	Xixin
DSD122	Cryptopin	369.45	2.38	0	6	2	78.74	0.72	Xixin
DSD123	sesamin	354.38	2.25	0	6	2	56.55	0.83	Xixin
DSD124	ZINC05223929	354.38	2.25	0	6	2	31.57	0.83	Xixin
GFD1	(-)-catechin	290.29	1.02	5	6	1	49.68	0.24	Dazao
GFD2	(+)-catechin	290.29	1.02	5	6	1	54.83	0.24	Dazao
GFD3	(S)-Coclaurine	285.37	2.2	3	4	3	42.35	0.24	Dazao
GFD4	21302-79-4	486.76	4.53	3	5	3	73.52	0.77	Dazao
GFD5	berberine	336.39	3.75	0	4	2	36.86	0.78	Dazao
GFD6	coumestrol	268.23	2.43	2	5	0	32.49	0.34	Dazao
GFD7	Fumarine	353.4	1.95	0	6	0	59.26	0.83	Dazao
GFD8	Jujubasaponin V_qt	472.78	4.61	2	4	2	36.99	0.63	Dazao
GFD9	jujuboside A_qt	472.78	3.8	2	4	1	36.67	0.62	Dazao
GFD10	Jujuboside C_qt	472.78	3.8	2	4	1	40.26	0.62	Dazao
GFD11	malkangunin	432.56	2.72	2	7	6	57.71	0.63	Dazao
GFD12	Mauritine D	342.46	1.11	2	6	2	89.13	0.45	Dazao
GFD13	Moupinamide	313.38	2.46	3	5	6	86.71	0.26	Dazao
GFD14	Nuciferin	295.41	3.38	0	3	2	34.43	0.4	Dazao
GFD15	quercetin	302.25	1.07	5	7	1	46.43	0.28	Dazao
GFD16	Ruvoside_qt	390.57	1.42	3	5	2	36.12	0.76	Dazao
GFD17	Spiradine A	311.46	1.29	1	3	0	113.52	0.61	Dazao
GFD18	stepharine	297.38	1.76	1	4	2	31.55	0.33	Dazao
GFD19	Stepholidine	327.41	2.26	2	5	2	33.11	0.54	Dazao
GFD20	Ziziphin_qt	472.78	3.8	2	4	1	66.95	0.62	Dazao
GFD21	zizyphus saponin I_qt	472.78	3.8	2	4	1	32.69	0.62	Dazao
GFD22	(-)-Medicocarpin	432.46	1.26	4	9	4	40.99	0.95	Gancao
GFD23	(2R)-7-hydroxy-2-(4-hydroxyphenyl)chroman-4-one	256.27	2.79	2	4	1	71.12	0.18	Gancao
GFD24	(2S)-6-(2,4-dihydroxyphenyl)-2-(2-hydroxypropan-2-yl)-4-methoxy-2,3-dihydrofuro[3,2-g]chromen-7-one	384.41	2.61	3	7	3	60.25	0.63	Gancao
GFD25	(2S)-7-hydroxy-2-(4-hydroxyphenyl)-8-(3-methylbut-2-enyl)chroman-4-one	324.4	3.62	2	4	3	36.57	0.32	Gancao
GFD26	(E)-1-(2,4-dihydroxyphenyl)-3-(2,2-dimethylchromen-6-yl)prop-2-en-1-one	322.38	4.46	2	4	3	39.62	0.35	Gancao
GFD27	(E)-3-[3,4-dihydroxy-5-(3-methylbut-2-enyl)phenyl]-1-(2,4-dihydroxyphenyl)prop-2-en-1-one	340.4	3.47	4	5	5	46.27	0.31	Gancao
GFD28	1,3-dihydroxy-8,9-dimethoxy-6-benzofurano[3,2-c]chromenone	328.29	2.74	2	7	2	62.9	0.53	Gancao
GFD29	1,3-dihydroxy-9-methoxy-6-benzofurano[3,2-c]chromenone	298.26	2.48	2	6	1	48.14	0.43	Gancao
GFD30	18α-hydroxyglycyrrhetic acid	486.76	4.54	3	5	1	41.16	0.71	Gancao
GFD31	1-Methoxyphaseollidin	354.43	3.66	2	5	3	69.98	0.64	Gancao
GFD32	2-(3,4-dihydroxyphenyl)-5,7-dihydroxy-6-(3-methylbut-2-enyl)chromone	354.38	2.99	4	6	3	44.15	0.41	Gancao
GFD33	2-[(3R)-8,8-dimethyl-3,4-dihydro-2H-pyrano[6,5-f]chromen-3-yl]-5-methoxyphenol	338.43	4.35	1	4	2	36.21	0.52	Gancao
GFD34	3-(2,4-dihydroxyphenyl)-8-(1,1-dimethylprop-2-enyl)-7-hydroxy-5-methoxy-coumarin	368.41	3.92	3	6	4	59.62	0.43	Gancao
GFD35	3-(3,4-dihydroxyphenyl)-5,7-dihydroxy-8-(3-methylbut-2-enyl)chromone	354.38	3.02	4	6	3	66.37	0.41	Gancao
GFD36	3’-Hydroxy-4’-O-Methylglabridin	354.43	3.76	2	5	2	43.71	0.57	Gancao
GFD37	3’-Methoxyglabridin	354.43	3.76	2	5	2	46.16	0.57	Gancao
GFD38	5,7-dihydroxy-3-(4-methoxyphenyl)-8-(3-methylbut-2-enyl)chromone	352.41	3.29	2	5	4	30.49	0.41	Gancao
GFD39	6-prenylated eriodictyol	356.4	2.99	4	6	3	39.22	0.41	Gancao
GFD40	7,2’,4’-trihydroxy-5-methoxy-3-arylcoumarin	300.28	2.51	3	6	2	83.71	0.27	Gancao
GFD41	7-Acetoxy-2-methylisoflavone	294.32	3.41	0	4	3	38.92	0.26	Gancao
GFD42	7-Methoxy-2-methyl isoflavone	266.31	3.48	0	3	2	42.56	0.2	Gancao
GFD43	8-(6-hydroxy-2-benzofuranyl)-2,2-dimethyl-5-chromenol	308.35	4.27	2	4	1	58.44	0.38	Gancao
GFD44	8-prenylated eriodictyol	356.4	2.99	4	6	3	53.79	0.4	Gancao
GFD45	Calycosin	284.28	2.82	2	5	2	47.75	0.24	Gancao
GFD46	dehydroglyasperins C	340.4	3.11	4	5	3	53.82	0.37	Gancao
GFD47	DFV	256.27	2.79	2	4	1	32.76	0.18	Gancao
GFD48	echinatin	270.3	3.41	2	4	4	66.58	0.17	Gancao
GFD49	Eurycarpin A	338.38	3.29	3	5	3	43.28	0.37	Gancao
GFD50	formononetin	268.28	3.01	1	4	2	69.67	0.21	Gancao
GFD51	Gancaonin A	352.41	3.34	2	5	4	51.08	0.4	Gancao
GFD52	Gancaonin B	368.41	3.14	3	6	4	48.79	0.45	Gancao
GFD53	Gancaonin G	352.41	3.25	2	5	4	60.44	0.39	Gancao
GFD54	Gancaonin H	420.49	3.99	3	6	3	50.1	0.78	Gancao
GFD55	Glabranin	324.4	3.59	2	4	3	52.9	0.31	Gancao
GFD56	Glabrene	322.38	3.68	2	4	1	46.27	0.44	Gancao
GFD57	Glabridin	324.4	3.81	2	4	1	53.25	0.47	Gancao
GFD58	Glabrone	336.36	3.78	2	5	1	52.51	0.5	Gancao
GFD59	Glepidotin A	338.38	2.88	3	5	3	44.72	0.35	Gancao
GFD60	Glepidotin B	340.4	2.88	3	5	3	64.46	0.34	Gancao
GFD61	glyasperin B	370.43	3.14	3	6	4	65.22	0.44	Gancao
GFD62	Glyasperin C	356.45	3.53	3	5	4	45.56	0.4	Gancao
GFD63	glyasperin F	354.38	3.52	3	6	1	75.84	0.54	Gancao
GFD64	Glyasperins M	368.41	3.57	2	6	2	72.67	0.59	Gancao
GFD65	Glycyrin	382.44	3.78	2	6	5	52.61	0.47	Gancao
GFD66	Glycyrol	366.39	4.06	2	6	3	90.78	0.67	Gancao
GFD67	Glycyrrhiza flavonol A	370.38	2.18	4	7	1	41.28	0.6	Gancao
GFD68	Glypallichalcone	284.33	3.8	1	4	5	61.6	0.19	Gancao
GFD69	Glyzaglabrin	298.26	2.32	2	6	1	61.07	0.35	Gancao
GFD70	HMO	268.28	2.92	1	4	2	38.37	0.21	Gancao
GFD71	Inermine	284.28	2.19	1	5	0	75.18	0.54	Gancao
GFD72	Inflacoumarin A	322.38	4.36	2	4	3	39.71	0.33	Gancao
GFD73	Isoglycyrol	366.39	4.15	1	6	1	44.7	0.84	Gancao
GFD74	Isolicoflavonol	354.38	2.92	4	6	3	45.17	0.42	Gancao
GFD75	isoliquiritigenin	256.27	3.04	3	4	3	85.32	0.15	Gancao
GFD76	isorhamnetin	316.28	1.31	4	7	2	49.6	0.31	Gancao
GFD77	Isotrifoliol	298.26	2.54	2	6	1	31.94	0.42	Gancao
GFD78	Jaranol	314.31	2.8	2	6	3	50.83	0.29	Gancao
GFD79	kaempferol	286.25	1.23	4	6	1	41.88	0.24	Gancao
GFD80	kanzonols W	336.36	3.97	2	5	1	50.48	0.52	Gancao
GFD81	Licoagrocarpin	338.43	3.94	1	4	3	58.81	0.58	Gancao
GFD82	Licoagroisoflavone	336.36	2.95	2	5	2	57.28	0.49	Gancao
GFD83	licochalcone a	338.43	4.74	2	4	6	40.79	0.29	Gancao
GFD84	Licochalcone B	286.3	3.17	3	5	4	76.76	0.19	Gancao
GFD85	licochalcone G	354.43	4.21	3	5	6	49.25	0.32	Gancao
GFD86	Licocoumarone	340.4	4.1	3	5	4	33.21	0.36	Gancao
GFD87	licoisoflavanone	354.38	3.54	3	6	1	52.47	0.54	Gancao
GFD88	Licoisoflavone	354.38	2.99	4	6	3	41.61	0.42	Gancao
GFD89	Licoisoflavone B	352.36	3.54	3	6	1	38.93	0.55	Gancao
GFD90	licopyranocoumarin	384.41	2.47	3	7	3	80.36	0.65	Gancao
GFD91	Licoricone	382.44	3.08	2	6	5	63.58	0.47	Gancao
GFD92	liquiritin	418.43	0.43	5	9	4	65.69	0.74	Gancao
GFD93	Lupiwighteone	338.38	3.23	3	5	3	51.64	0.37	Gancao
GFD94	Medicarpin	270.3	3.07	1	4	1	49.22	0.34	Gancao
GFD95	naringenin	272.27	2.47	3	5	1	59.29	0.21	Gancao
GFD96	Odoratin	314.31	2.81	2	6	3	49.95	0.3	Gancao
GFD97	Phaseol	336.36	4.59	2	5	2	78.77	0.58	Gancao
GFD98	Phaseolinisoflavan	324.4	3.77	2	4	1	32.01	0.45	Gancao
GFD99	Pinocembrin	256.27	2.85	2	4	1	64.72	0.18	Gancao
GFD100	Quercetin der.	330.31	2.55	3	7	3	46.45	0.33	Gancao
GFD101	Semilicoisoflavone B	352.36	3.55	3	6	1	48.78	0.55	Gancao
GFD102	shinpterocarpin	322.38	4.13	1	4	0	80.3	0.73	Gancao
GFD103	Sigmoidin-B	356.4	3.02	4	6	3	34.88	0.41	Gancao
GFD104	Vestitol	272.32	2.89	2	4	2	74.66	0.21	Gancao
GFD105	ent-Epicatechin	290.29	2.83	5	6	1	48.96	0.24	Guizhi
GFD106	beta-sitosterol	414.79	3.2	1	1	6	36.91	0.75	Guizhi
GFD107	sitosterol	414.79	2.71	1	1	6	36.91	0.75	Guizhi
GFD108	(-)-taxifolin	304.27	1.66	5	7	1	60.51	0.27	Guizhi
GFD109	DMEP	282.32	1.93	0	6	10	55.66	0.15	Guizhi
GFD110	6-gingerol	294.43	3.45	2	4	10	35.64	0.16	Shengjiang
GFD111	6-shogaol	276.41	4.95	1	3	9	31	0.14	Shengjiang
GFD112	(R)-Norcoclaurine	271.34	1.73	4	4	2	82.54	0.21	Fuzi
GFD113	6-Demethyldesoline	453.64	-0.53	4	8	5	51.87	0.66	Fuzi
GFD114	benzoylnapelline	463.67	2.9	2	5	4	34.06	0.53	Fuzi
GFD115	Deltoin	328.39	2.95	0	5	4	46.69	0.37	Fuzi
GFD116	Deoxyandrographolide	334.5	2.71	2	4	4	56.3	0.31	Fuzi
GFD117	ignavine	449.59	0.72	3	6	3	84.08	0.25	Fuzi
GFD118	isotalatizidine	407.61	0.41	3	6	4	50.82	0.73	Fuzi
GFD119	karakoline	377.58	0.8	3	5	2	51.73	0.73	Fuzi
GFD120	Karanjin	292.3	3.42	0	4	2	69.56	0.34	Fuzi
HGWD1	(+)-catechin	290.29	1.02	5	6	1	54.83	0.24	Baishao
HGWD2	(3S,5R,8R,9R,10S,14S)-3,17-dihydroxy-4,4,8,10,14-pentamethyl-2,3,5,6,7,9-hexahydro-1H-cyclopenta[a]phenanthrene-15,16-dione	358.52	3.52	2	4	0	43.56	0.53	Baishao
HGWD3	11alpha,12alpha-epoxy-3beta-23-dihydroxy-30-norolean-20-en-28,12beta-olide	470.71	3.82	2	5	1	64.77	0.38	Baishao
HGWD4	albiflorin_qt	318.35	0.53	2	6	4	66.64	0.33	Baishao
HGWD5	kaempferol	286.25	1.23	4	6	1	41.88	0.24	Baishao
HGWD6	Lactiflorin	462.49	0.31	3	10	5	49.12	0.8	Baishao
HGWD7	paeoniflorgenone	318.35	0.86	1	6	4	87.59	0.37	Baishao
HGWD8	paeoniflorin_qt	318.35	0.69	2	6	4	68.18	0.4	Baishao
HGWD9	(-)-catechin	290.29	1.02	5	6	1	49.68	0.24	Dazao
HGWD10	(S)-Coclaurine	285.37	2.2	3	4	3	42.35	0.24	Dazao
HGWD11	21302-79-4	486.76	4.53	3	5	3	73.52	0.77	Dazao
HGWD12	berberine	336.39	3.75	0	4	2	36.86	0.78	Dazao
HGWD13	coumestrol	268.23	2.43	2	5	0	32.49	0.34	Dazao
HGWD14	Fumarine	353.4	1.95	0	6	0	59.26	0.83	Dazao
HGWD15	Jujubasaponin V_qt	472.78	4.61	2	4	2	36.99	0.63	Dazao
HGWD16	jujuboside A_qt	472.78	3.8	2	4	1	36.67	0.62	Dazao
HGWD17	Jujuboside C_qt	472.78	3.8	2	4	1	40.26	0.62	Dazao
HGWD18	malkangunin	432.56	2.72	2	7	6	57.71	0.63	Dazao
HGWD19	Mauritine D	342.46	1.11	2	6	2	89.13	0.45	Dazao
HGWD20	Moupinamide	313.38	2.46	3	5	6	86.71	0.26	Dazao
HGWD21	Nuciferin	295.41	3.38	0	3	2	34.43	0.4	Dazao
HGWD22	quercetin	302.25	1.07	5	7	1	46.43	0.28	Dazao
HGWD23	Ruvoside_qt	390.57	1.42	3	5	2	36.12	0.76	Dazao
HGWD24	Spiradine A	311.46	1.29	1	3	0	113.52	0.61	Dazao
HGWD25	stepharine	297.38	1.76	1	4	2	31.55	0.33	Dazao
HGWD26	Stepholidine	327.41	2.26	2	5	2	33.11	0.54	Dazao
HGWD27	Ziziphin_qt	472.78	3.8	2	4	1	66.95	0.62	Dazao
HGWD28	zizyphus saponin I_qt	472.78	3.8	2	4	1	32.69	0.62	Dazao
HGWD29	ent-Epicatechin	290.29	2.83	5	6	1	48.96	0.24	Guizhi
HGWD30	beta-sitosterol	414.79	3.2	1	1	6	36.91	0.75	Guizhi
HGWD31	sitosterol	414.79	2.71	1	1	6	36.91	0.75	Guizhi
HGWD32	(-)-taxifolin	304.27	1.66	5	7	1	60.51	0.27	Guizhi
HGWD33	DMEP	282.32	1.93	0	6	10	55.66	0.15	Guizhi
HGWD34	6-gingerol	294.43	3.45	2	4	10	35.64	0.16	Shengjiang
HGWD35	6-shogaol	276.41	4.95	1	3	9	31	0.14	Shengjiang
HGWD36	(6aR,11aR)-9,10-dimethoxy-6a,11a-dihydro-6H-benzofurano[3,2-c]chromen-3-ol	300.33	2.88	1	5	2	64.26	0.42	Huangqi
HGWD37	1,7-Dihydroxy-3,9-dimethoxy pterocarpene	314.31	2.85	2	6	2	39.05	0.48	Huangqi
HGWD38	3,9-di-O-methylnissolin	314.36	3.28	0	5	3	53.74	0.48	Huangqi
HGWD39	7-O-methylisomucronulatol	316.38	2.85	1	5	4	74.69	0.3	Huangqi
HGWD40	Bifendate	418.38	1.75	0	10	7	31.1	0.67	Huangqi
HGWD41	Calycosin	284.28	2.82	2	5	2	47.75	0.24	Huangqi
HGWD42	formononetin	268.28	3.01	1	4	2	69.67	0.21	Huangqi
HGWD43	isoflavanone	316.33	2.76	2	6	3	109.99	0.3	Huangqi
HGWD44	isorhamnetin	316.28	1.31	4	7	2	49.6	0.31	Huangqi
HGWD45	Jaranol	314.31	2.8	2	6	3	50.83	0.29	Huangqi
HGWD46	9,10-dimethoxypterocarpan-3-O-β-D-glucoside	462.49	1.18	4	10	5	36.74	0.92	Huangqi
HGWD47	(Z)-1-(2,4-dihydroxyphenyl)-3-(4-hydroxyphenyl)prop-2-en-1-one	256.27	3.04	3	4	3	87.51	0.15	Huangqi
HGWD48	(3R)-3-(2-hydroxy-3,4-dimethoxyphenyl)chroman-7-ol	302.35	2.76	2	5	3	67.67	0.26	Huangqi

### C-T Network Construction

To facilitate analysis of the complex relationships between active components and their targets of three formulas, component-target networks were constructed by using Cytoscape (**Figures S1–S3**). The results revealed that the DSD network consisted of 124 active components, 846 target proteins, and 3758 interactions; the GFD network contained120 active components, 821 target proteins, and 3759 interactions; the HGWD network consisted of 48 active components, 612 target proteins, and 1373 interactions.

We further analyzed the topology parameters of these C-T networks using NetworkAnalyzer and found that the average degree of components and targets in DSD were 30.31 and 5.20; the average degree of components and targets in GFD were 31.33 and 5.36; the average degree of components and targets in HGWD were 28.6 and 2.43. These results indicate that there exist interactions between one component and multiple targets in three formulas, and also exist phenomenon that different components act on the same target, which is in line with the characteristics of multi-component and multi-target mediated synergistic effect of TCM, and also reflects the complexity of the mechanism of TCM.

### KNMSs Predication and Validation

#### KNMSs Predication

**Figure 3:**
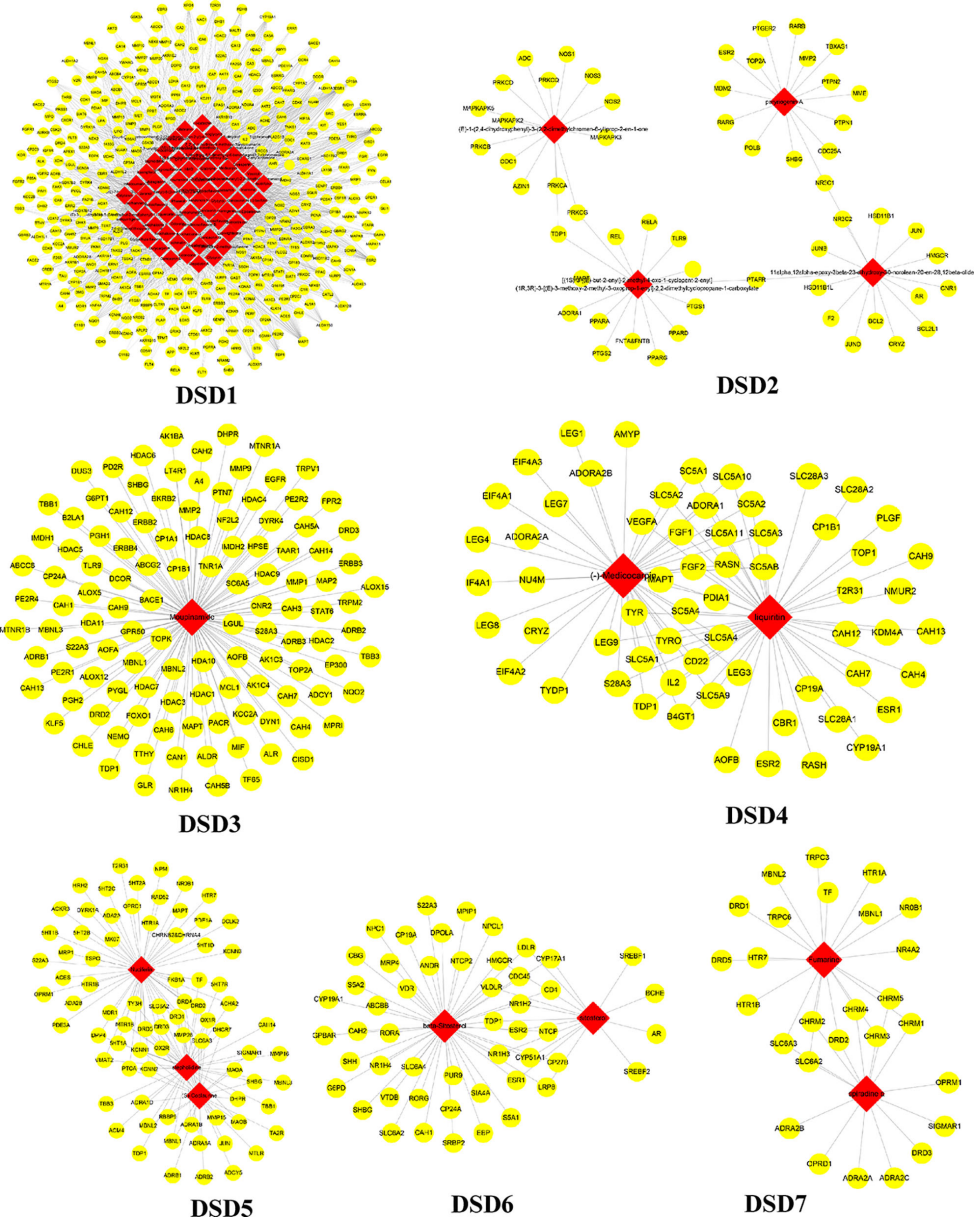
The predicated KNMSs of C-T network of DSD. The red nodes represent the specific components of DSD, and the yellow nodes represent related targets.

**Figure 4:**
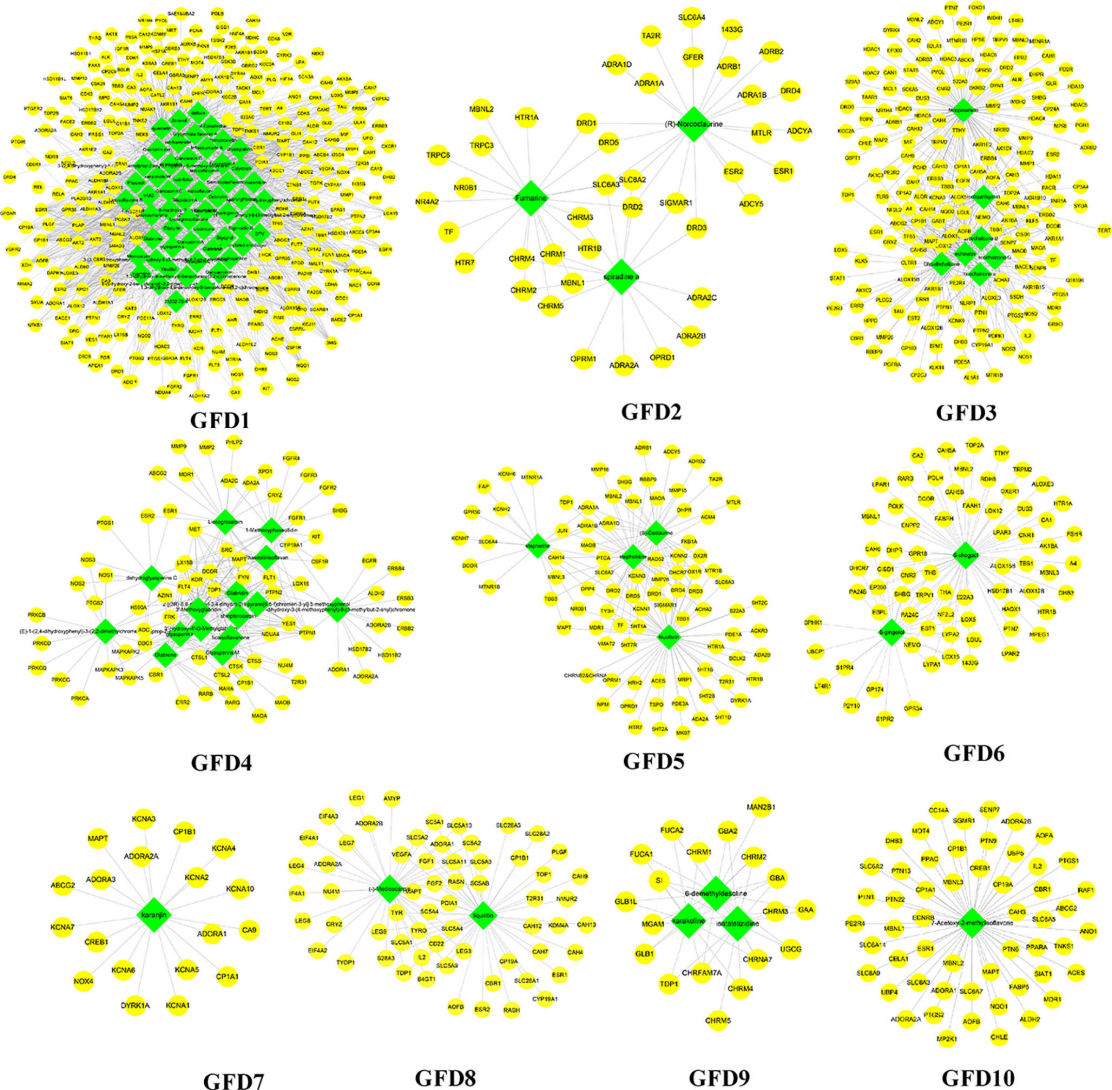
The predicated KNMSs of C-T network of GFD. The green nodes represent the specific components of GFD, and the yellow nodes represent related targets.

**Figure 5:**
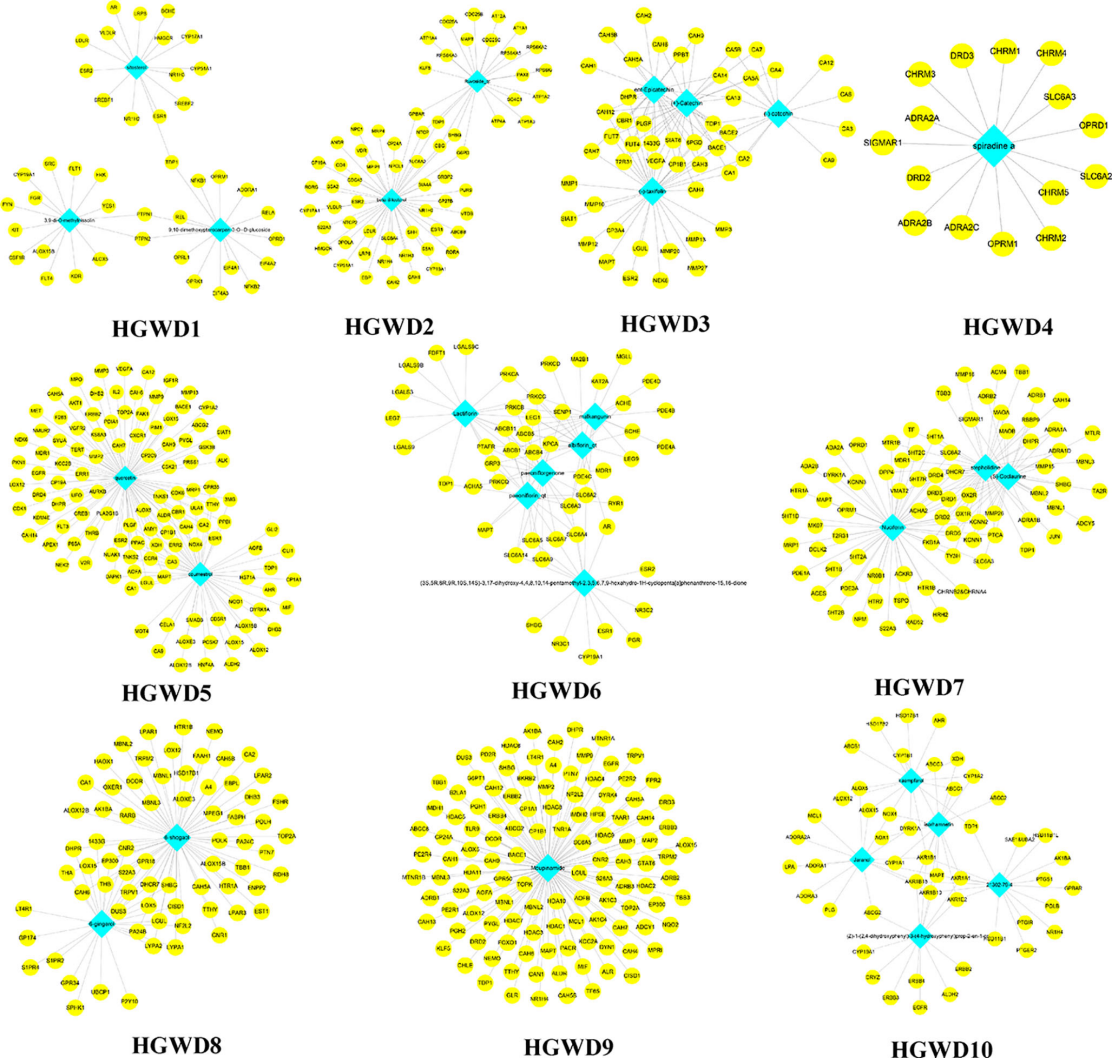
The predicated KNMSs of C-T network of HGWD. The blue nodes represent the specific components of HGWD, and the yellow nodes represent related targets.

These C-T networks are complex and huge. How to quickly extract important information from these complex networks is the key step to decode underlying molecular mechanism. Here, we introduced the infomap algorithm in the network pharmacology model for the first time based on the random walk theory combined with Huffman-encoding. The algorithm performs to optimize the discovery of KNMSs in C-T network heuristically by using a reasonable global metric. 7, 10, and 10 KNMSs were predicted in DSD, GFD, and HGWD, respectively (p value < 0.05) (**Figures 3**–**5**). The detail information of network KNMSs were shown in **Table S2**.

#### KNMSs Validation

In order to validate whether predicted KNMSs in each formula can represent corresponding full C-T networks in treating RA. Three strategies were used to verify the accuracy and reliability and of KNMSs. The first strategy was used to see whether the number of RA pathogenic genes in KNMSs are close to the number of RA pathogenic genes in CT network. The coverage was defined as the percentage of the number of pathogenic genes in KNMSs to the number of pathogenic genes in C-T network. High coverage indicated that KNMSs could retain most formula-targeted RA pathogenic genes that included in the corresponding C-T network. The second strategy was designed to see whether the gene enrichment pathways in KNMSs covers the gene enrichment pathways in C-T network as much as possible. High coverage indicated that KNMSs could cover most genes enriched pathways of the corresponding C-T network. The third strategy was employed to calculate the percentage of cumulative contribution of important nodes in KNMSs to that of nodes in C-T network. High percentage means KNMSs can retain the important nodes in the corresponding C-T network. The detail results are as follows:

##### Validated the Number and Coverage of Pathogenic Genes in KNMSs

**Figure 6:**
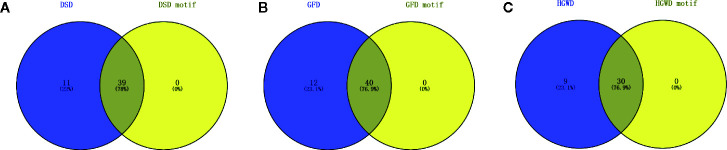
The number of overlap pathogenic genes between C-T network and KNMSs in DSD, GFD and HGWD **(A–C)**. **(A–C)** use venn diagram to visualize the overlap number between C-T network and KNMSs in DSD, GFD, and HGWD, respectively.

To assess whether the number of RA pathogenic genes in KNMSs are close to the number of RA pathogenic genes in corresponding C-T network. The known pathogenic genes of RA reported by published literature and databases were collected, and the pathogenic genes confirmed by more than 5 literatures were selected for further analysis (**Table S3**). We found that the C-T network of DSD, GFD, and HGWD contains 50, 52, and 39 pathogenic genes, respectively. While the KNMSs of DSD, GFD, and HGWD contains 39, 40, and 30 pathogenic genes. The number of pathogenic genes in KNMSs compared to that in C-T network of DSD, GFD, and HGWD reached 78%, 76.9%, and 76.9%, which confirmed that the predicted KNMSs with high coverage of pathogenic genes (**Figures 6A–C**). These results demonstrate that KNMSs have a high coincidence degree with C-T network in the number and coverage of pathogenic genes, it also indicated that our proposed KNMS detection model can maximize the coincidence degree of pathogenic genes in the C-T network of formulas.

##### Validated the Genes Enriched Pathways in KNMSs

**Figure 7:**
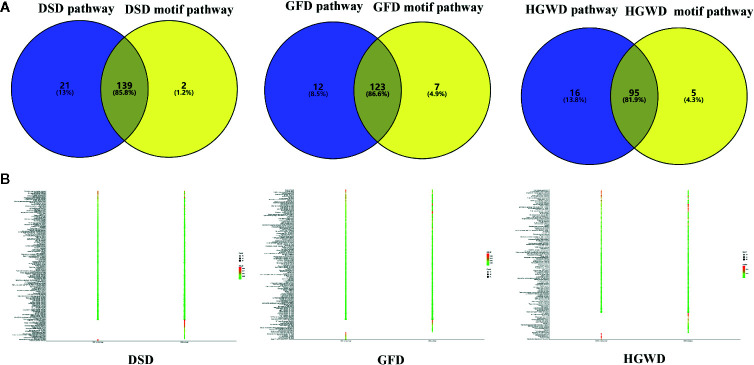
The functional similarity analysis between C-T network and KNMSs in DSD, GFD, and HGWD, respectively. **(A, B)** represent the functional similarity visualized by venn diagram and bubble diagram, respectively.

An additional metric for evaluating the importance of the inferred motifs is determined by their functional coherence, which can be accessed *via* their related genes enrichment pathways from KEGG (Kanehisa and Goto, 2000). Here, we used this method to detect whether KNMSs found in each formula can represent their full C-T networks at functional level. Our analysis shown that genes enriched pathways of KNMSs in DSD accounts for 85.8% of genes enriched pathways of the full C-T network in DSD; genes enriched pathways of KNMSs in GFD accounts for 86.6% genes enriched pathways of the full C-T network in GFD; genes enriched pathways of KNMSs in HGWD accounts for 81.9% genes enriched pathways of the full C-T network in HGWD (**Figures 7A, B**). It was encouraged that the gene enriched pathways involved in KNMSs of 3 formulas are highly compatible with gene enriched pathways of their C-T networks. This result confirmed that KNMSs have a high coincidence degree with C-T network at the gene functional level and also suggested that our proposed KNMS detection model can maximize the retention of functional pathways in the formulas of TCM.

##### Validated the Cumulative Contribution of Important Nodes in KNMSs

**Figure 8:**
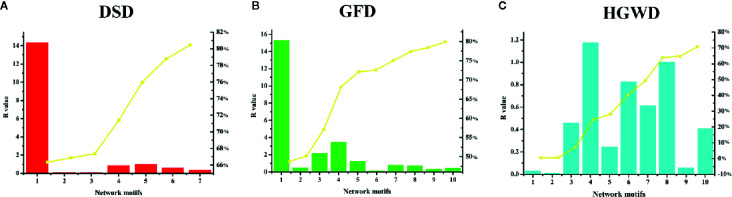
The contribution coefficient of network topological between C-T network and KNMSs in DSD, GFD and HGWD **(A–C)**. **(A–C)** use bar diagram to visualize the cumulative contribution rate between C-T network and KNMSs in DSD, GFD and HGWD, respectively.

The degree of nodes is a key topological parameter that characterizes the influence of nodes in a network (Lv et al., 2014). Here, a mathematical model was established to evaluate the importance of KNMSs in each formula based on the degree of nodes. According to the calculation results, each KNMS was assigned a CC value. The detailed information was shown in **Figure 8** and **Table S4**. The sum of CC of 7, 10, and 10 KNMSs in each formula reached 80.44%, 79.88%, and 70.76% of that in C-T networks of DSD, GFD, and HGWD, respectively. The results confirmed that KNMSs have a high coincidence degree with C-T network on the topological structure and also indicated that our proposed KNMS detection model could maximize the coverage of important network topological structures compared with C-T network in each formula.

### Potential Mechanisms Analysis of Different Formulas Treats the Same Disease

**Figure 9:**
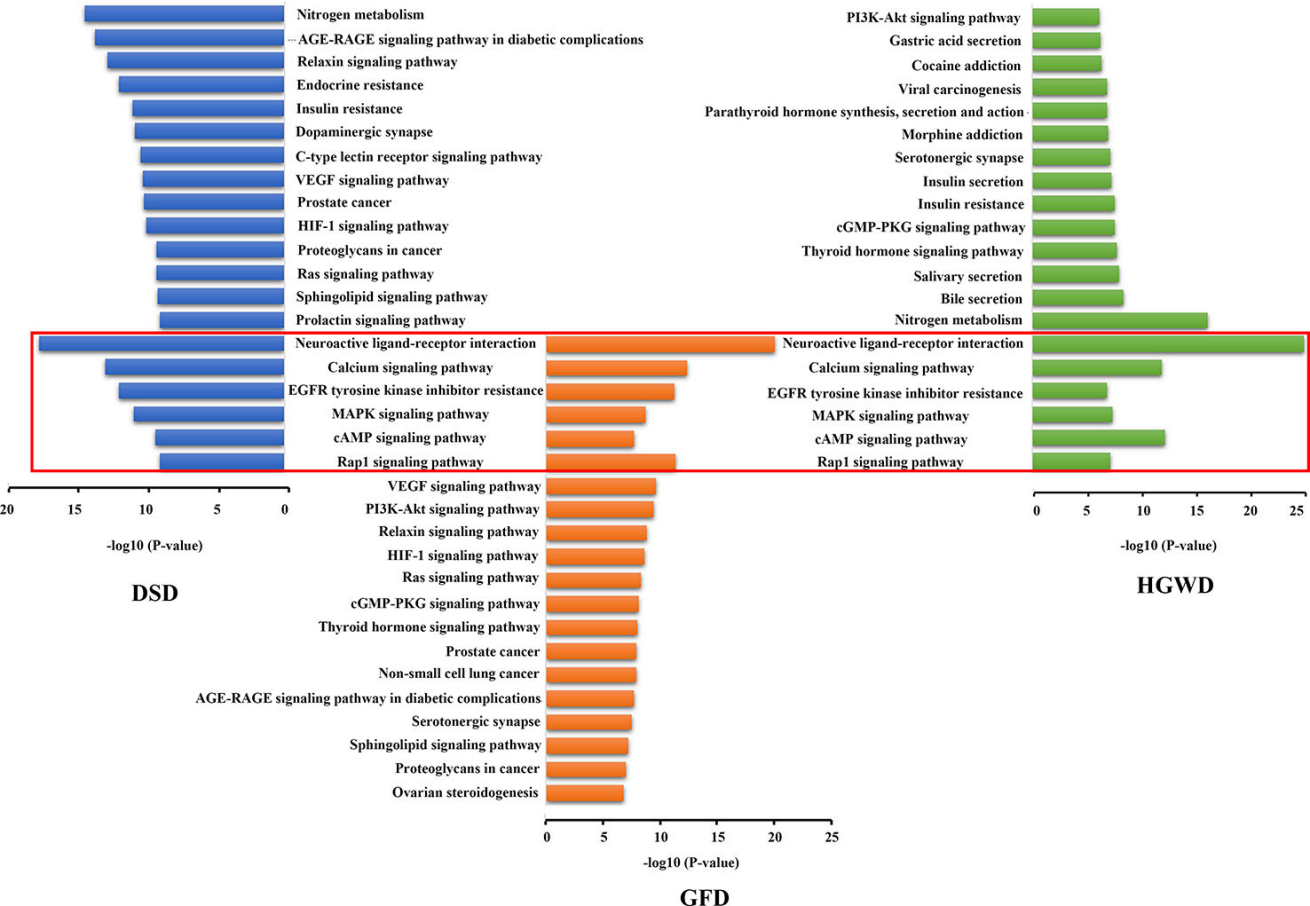
The enrichment pathway map of KNMSs in the C-T network of DSD, GFD, and HGWD.

In order to reveal the potential mechanism of KNMSs in different formula for treating rheumatoid arthritis, pathway enrichment analysis of KNMS-related genes in each formula were performed. In the DSD, genes in total 7 KNMSs were enriched in 165 pathways, genes in two KNMSs, DSD1, and DSD2 were enriched in 158 pathways, accounting for 95.8% of that in 7 KNMSs. The arthritis-related signaling pathways corresponding to DSD1 and DSD2 were partially complementary, for example, genes in DSD1 were mainly enriched in JAK-STAT signaling pathway and AMPK signaling pathway, genes in DSD2 mainly enriched in NF-kappa B signaling pathway, p53 signaling pathway and Wnt signaling pathway. In GFD, we total got 10 KNMSs. Genes in these 10 KNMSs were enriched in 151 pathways. Four of 10 KNMSs, GFD1, GFD3, GFD4, and GFD5 related genes are enriched in 144 pathways, accounting for 95.4% of enrichment pathways in 10 KNMSs of GFD. Moreover, some of their corresponding arthritis-related signaling pathways are complementary. For example, GFD1 mainly includes TNF signaling pathway, IL-17 signaling pathway and AMPK signaling pathway, and GFD3 mainly includes Inflammatory mediator regulation of TRP channels and GnRH signaling pathway. In HGWD, genes in 10 predicted KNMSs were enriched in 110 pathways, genes in HGWD4 and HGWD5, HGWD6, and HGWD8 covered 102 pathways, accounting for 92.7% of all KNMSs gene enrichment pathways. Consistent with DSD and GFD results, some of their corresponding arthritis-related signal pathways were also complementary. For example, HGWD4 mainly includes TNF signaling pathway, Hedgehog signaling pathway and IL-17 signaling pathway, HGWD5 mainly includes VEGF signaling pathway, NF-kappa B signaling pathway and mTOR signaling pathway, HGWD6 mainly includes cAMP signaling pathway and cGMP-PKG signaling pathway (**Figure 9**, **Table S5**). These results show that KNMSs in different formulas have distinct roles and synergistic effects in the treatment of rheumatoid arthritis.

**Figure 10:**
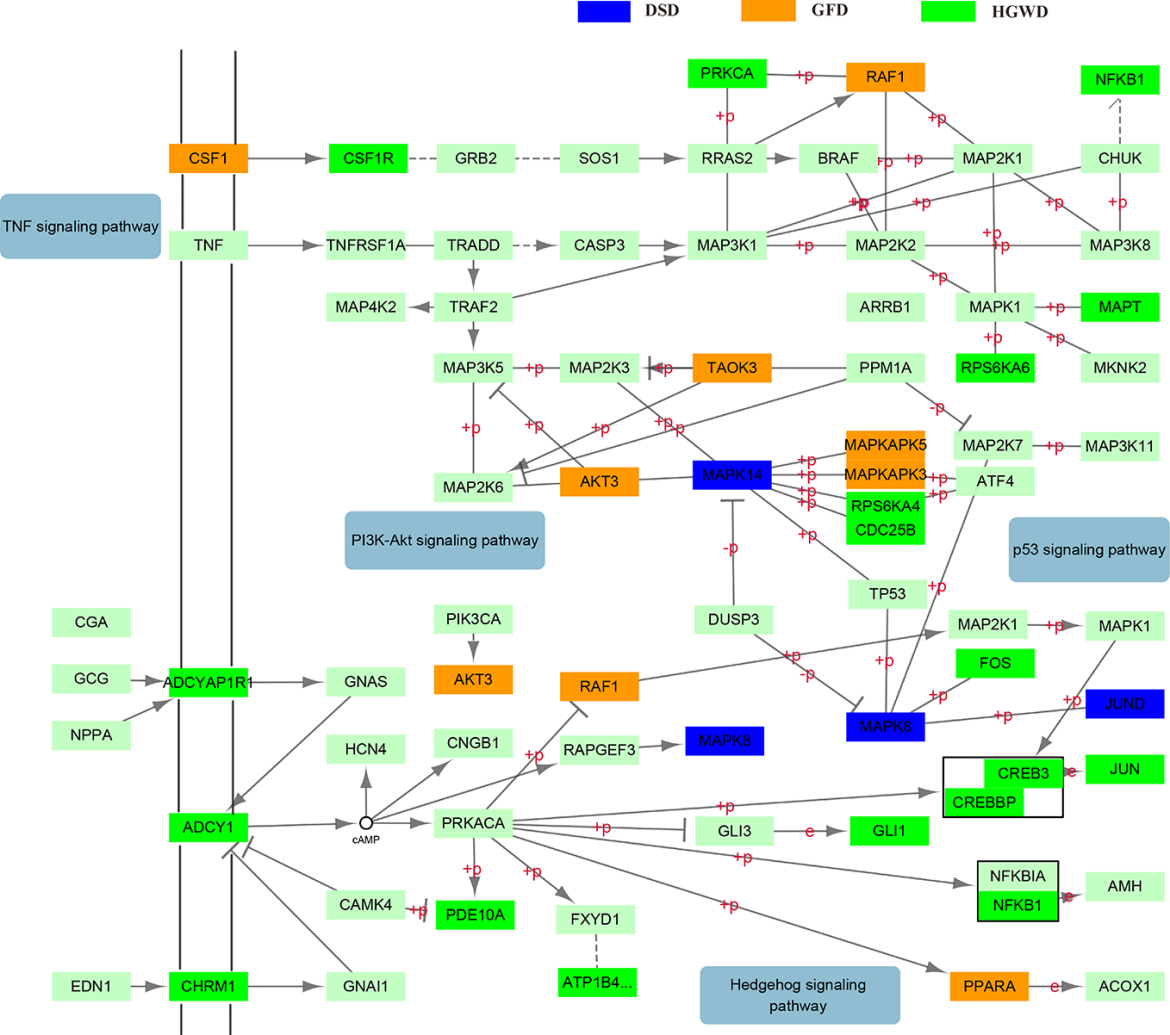
Gene enrichment analysis of all KNMSs from DSD, GFD, and HGWD, respectively.

In order to further explore the potential mechanism of the three formulas in treating RA, besides the difference analysis of each KNMS in different formulas, KEGG enrichment analysis of all KNMSs in each formula were also implemented and found that 3 formulas play the therapeutic effect on RA through the following five common pathways: Rap1 signaling pathway, cAMP signaling pathway, MAPK signaling pathway, EGFR Tyrosine Kinase Inhibitor Resistance, Calcium signaling pathway and Neuroactive ligand-receptor interaction. Except the common pathways, we found that the three formulas can play the role of treating RA through their specific pathways (**Figure 10**). For example, DSD can play the role of treating RA by regulating VEGF signaling pathway. GFD can play a role in treating RA by regulating HIF-1 signaling pathway. HGWD can play a role in treating RA by regulating PI3K-Akt signaling pathway. These results indicate that 3 formulas can play the role of treating RA through different and common pathways, which may act as the essence of different formulas treat the same disease.

**Figure 11:**
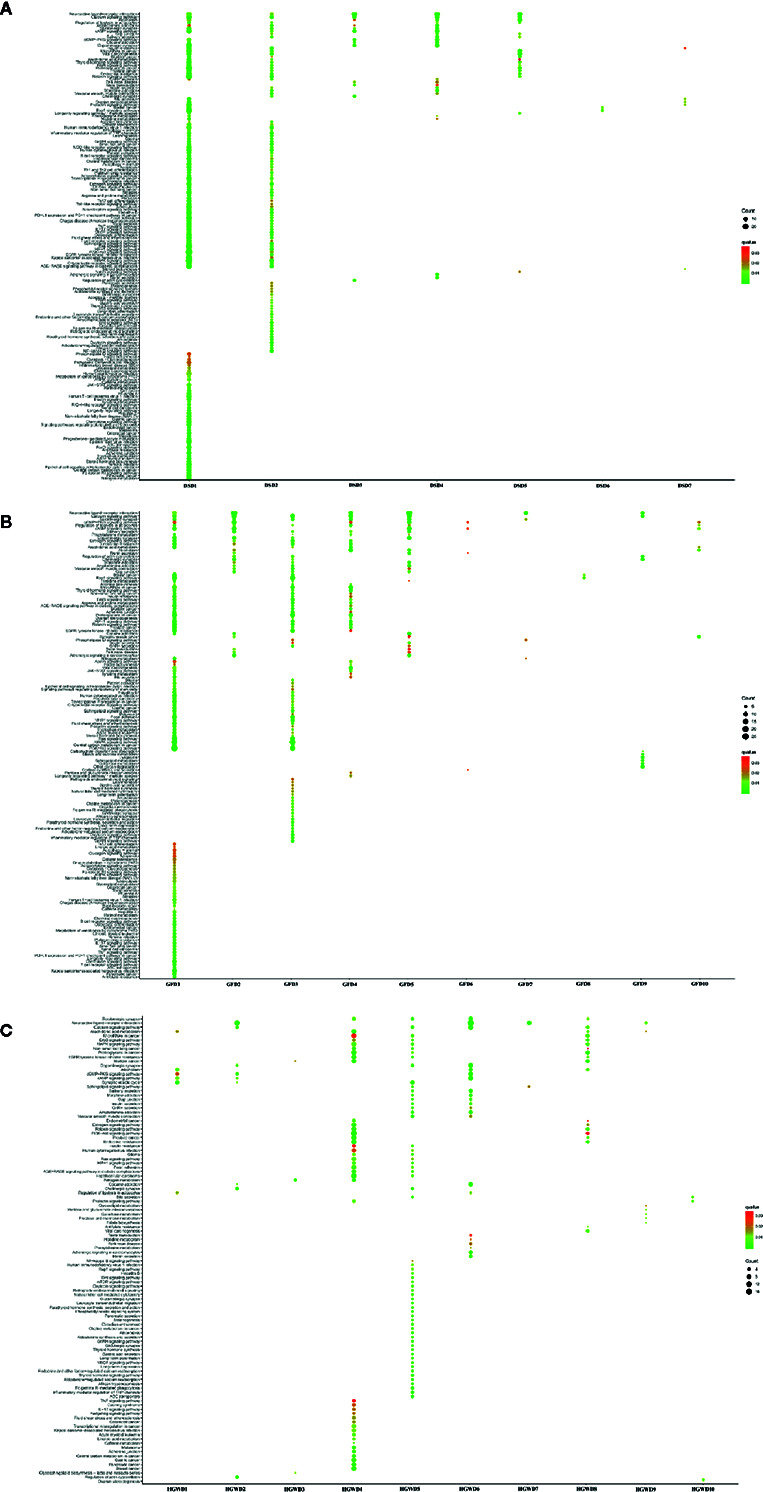
Distribution of specific targets enriched pathways of KNMSs in three formulas on the comprehensive inflammatory pathway. **(A–C)** represent the distribution of targets enriched pathways of KNMSs in DSD, GFD and HGWD on the comprehensive inflammatory pathway, respectively.

Through PubMed literature search, we found that among the common pathways, MAPK signaling pathway and cAMP signaling pathway have the most correlation records with rheumatoid arthritis. We selected MAPK signaling pathway and cAMP signaling pathway which were reported closely related to inflammation to illustrate the mechanism of different formulas treat the same disease in detail. Firstly, a comprehensive inflammatory pathway was constructed by integrating the two pathways. And then, the genes in KNMSs of three formulas were mapped to the comprehensive inflammatory pathway (**Figure 11**). Results show that genes in the KNMSs of DSD mainly distributed in the downstream of the comprehensive inflammatory pathway, such as MAPK14, MAPK8, and JUND; Genes in the KNMSs of GFD mainly distributed in the downstream of the comprehensive inflammatory pathway, such as AKT3, RAF1, and TAOK3; while genes in the KNMSs of HGWD distributed both in the upstream and downstream of the comprehensive inflammatory pathway, such as CSF1R, ADCYAP1R1, CHRM1, NFKB1, MAPT, and JUN. The results suggest that different formulas play therapeutic roles through targeting different genes in the comprehensive inflammatory pathway.

### Experimental Validation *In Vitro*

**Figure 12:**
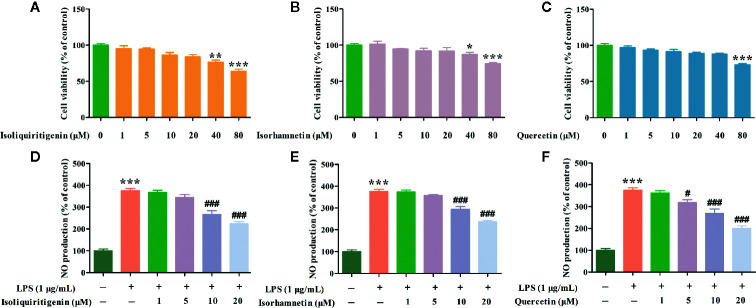
Effects of isoliquiritigenin **(A, D)**, isorhamnetin **(B, E)**, and quercetin **(C, F)** on cell viabilities and NO of LPS induced RAW264.7 cells. ^*^p < 0.05, ^**^p < 0.01, ^***^p < 0.001 compared with control group. ^#^p < 0.05, ^###^p < 0.001 compared with the LPS group.

Effects of isoliquiritigenin, isorhamnetin and quercetin with different concentrations on cell viabilities of RAW264.7 cells were detected by MTT assay. Compared with control group, 1, 5, 10, and 20 μM isoliquiritigenin, isorhamnetin, and quercetin had no effects on RAW264.7 cells viabilities (**Figures 12A–C**). Therefore, four concentrations were used (1, 5, 10, and 20 μM) for subsequent experiments.

NO is a regulator of information transmission between cells and has the function of mediating cellular immune and inflammatory reactions. In order to further evaluate the results obtained by the network pharmacology model, the key components in KNMSs of each formula were selected for experimental validation. Isoliquiritigenin from motif 1 (DSD1) of DSD, isorhamnetin from motif 1 (GFD1) of GFD, and quercetin from motif 5 (HGWD5) of HGWD were chose to detect potential anti-inflammatory effects using LPS induced RAW264.7 cells. Compared with control group, the NO level was significantly increased by 275.34% in the culture medium of LPS treated cells, however, isoliquiritigenin (10 and 20 μM) markedly decreased the extracellular NO levels by 107.94% and 151.04%, isorhamnetin (10 and 20 μM) markedly decreased the extracellular NO levels by 81.59% and 137.94%, quercetin (5, 10 and 20 μM) markedly decreased the extracellular NO levels by 56.68%, 106.57% and 174.59%, respectively, in a concentration-dependent manner (**Figures 12D–F**). Our results demonstrated that isoliquiritigenin, isorhamnetin, and quercetin inhibited NO production in LPS induced RAW264.7 cells.

## Discussion

The therapeutic effect of current synthetic agents in treating RA is not satisfactory and most of them have undesirable side effects. In China, some classical formulas have a long history of clinical application to treat RA and have shown significant curative effects. However, TCM formulas is a multi-component and multi-target agent from the molecular perspective (Corson and Crews, 2007; Bo et al., 2013). Based on the characteristics of complex components and unclear targets of TCM formula, the development of novel methods became an urgent issue needed to be solved.

TCM network pharmacology emerging recently has become a flourishing field in TCM modern studies along with the rapid progress of bioinformatics (Guo et al., 2017; Gao et al., 2018; Wang et al., 2018). So, using the method, combined with the rich experience of TCM treatment, could hopefully decode the underlying mechanism of TCM formula in the treatment of complex disease with the characteristic of “multi-targets, multi-component”. Network pharmacology approach could help us search for putative active components and targets of herbs based on widely existing databases and shows the network of drug-targets by a visual way (Gao et al., 2016). Moreover, it abstracts the interaction between drugs and target genes into a network model and investigates the effects of drugs on biological networks from a holistic perspective. It can help us to further understand potential action mechanisms of TCM within the context of interactions at the system level. However, in the decode process of complex networks, there are still exist redundancies and noises in current network pharmacology study.

In order to solve this problem, we introduced the infomap algorithm based on huffman encoding and the random walk theory for the first time. The algorithm performs to optimize the discovery of motif in C-T network heuristically by using a reasonable global metric. The results of optimized KNMSs are used to analyze the mechanism of different formulas for the treatment of RA. During this process the contribution coefficient model was used to validate the predicted KNMSs, which confirm the accuracy and reliability of our proposed strategy.

In this study, 230 active components of three formulas were found in total after ADME screening, 31 of these components are common to the three formulas, and 93, 89, and 17 components are specific to each formula. It suggested that the three formulas play therapeutic effect on rheumatoid arthritis through both common and specific components. In order to analyze the key component groups and mechanisms of the three formulas in the treatment of rheumatoid arthritis, we used target prediction tools to predict the targets of active components in different formulas and construct C-T networks. The degree distribution in the C-T network shows that the same components could act on different targets, and different components could also act on the same targets, which fully reflects the multi-component and multi-target complexity of TCM in treating complex diseases.

In order to quickly extract important information from complex C-T networks, motif prediction and validation strategy were used to rapidly discover the KNMSs of different formulas in the treatment of RA by using multidimensional data. More and more evidences show that network motif is an effective method to extract functional units and find core elements in complex networks. Radicchi et al. has confirmed that network motif offers an effective and manageable approach for characterizing rapidly the main functional unit of disease progression (Radicchi et al., 2004). Yang has reported that identifying overlapping motifs is crucial for understanding the structure as well as the function of real-world networks (Yang and Leskovec, 2012). Cai et al. indicate that uncovering motif structures of a complex network can help us to understand how the network play functions (Cai et al., 2014). Utilizing the network motif prediction model, 7, 10, and 10 KNMSs (p<0.05) were predicted in DSD, GFD and HGWD, respectively.

Coverage of RA pathogenic genes, coverage of functional pathways and cumulative contribution of key nodes were employed to evaluate the accuracy and reliability of KNMSs. The verification results show that KNMSs has a high coincidence degree with C-T network at the pathogenic genes, gene functional and topological structure level. It suggests that our proposed KNMS detection model can maximize the retention of functional pathways, the coverage of network topological structure and the coincidence degree of pathogenic genes in the formulas of TCM.

Through the analysis of KNMSs gene enrichment pathways in different formulas, we found that the percentage of gene enrichment pathways of different KNMSs is distinct compare to the gene enrichment pathways of all KNMSs in each formula. In DSD, gene enrichment pathways of DSD1, DSD2 account for more than 95% of gene enrichment pathways of 7 KNMSs. In GFD, the gene enrichment pathways of 4 KNMSs, GFD1, GFD3, GFD4, and GFD5 account for 95.4% of gene enrichment pathways of 10 KNMSs. In HGWD, the gene enrichment pathways of 4 KNMSs, HGWD4, HGWD5, HGWD6, and HGWD8 account for 92.7% of gene enrichment pathways of 10 KNMSs. These KNMSs in each formula play different roles by targeting on common and complementary inflammation-related signaling pathways. These complementary inflammatory signaling pathways include: DSD1 specifically related JAK-STAT signaling pathway and AMPK signaling pathway, DSD2 specifically related NF-kappa B signaling pathway, p53 signaling pathway and Wnt signaling pathway. GFD1 specifically related TNF signaling pathway, IL-17 signaling pathway, GFD3 specifically related Inflammatory mediator regulation of TRP channels and GnRH signaling pathway, HGWD4 specifically related TNF signaling pathway, Hedgehog signaling pathway, HGWD5 specifically related VEGF signaling pathway and mTOR signaling pathway, HGWD6 specifically related cAMP signaling pathway and cGMP-PKG signaling pathway. These results indicate that KNMSs in different formulas have distinct roles and synergistic effects in the treatment of rheumatoid arthritis.

In addition to the difference analysis of each KNMS in different formulas, KEGG enrichment analysis of all KNMSs in each formula were also implemented and revealed that 3 formulas exert the therapeutic effect of RA through common pathway, such as MAPK signaling pathway, cAMP signal pathway etc. or specific pathway, such as VEGF signaling pathway, HIF-1 signaling pathway, PI3K-Akt signaling pathway etc. Among them, MAPK signaling pathway plays an important role in the pathological process of RA (Schett and Zwerina, 2008). Its over-activation is closely related to inflammatory hyperplasia of synovial tissue and destruction of articular cartilage tissue. As an inducible transcription factor, MAPK regulates the expression of many genes and has been considered as a promising target for the treatment of RA (Rubbert-Roth, 2012). Studies have shown that collagen-induced arthritis rats administrated with MAPK signal transduction pathway inhibitor have significant differences in inhibiting synovitis, bone destruction and articular cartilage destruction compared with the group without signal pathway inhibitor (Adelheid et al., 2014). cAMP signal pathway is an important signal pathway for peripheral blood lymphocytes of RA patients. The study found that the cAMP level in peripheral blood lymphocytes (PBL) of RA patients increased, and its proliferation response was significantly lower than that of PBL in normal patients. It was also found that the abnormal activation of adenylate cyclase in RA patients was related to the low function of Gi protein (Dai and Wei, 2003). The formation of RA neovascularization depends on the expression of various angiogenic factors, especially VEGF and its receptor in RA (Mi-La et al., 2006). It has been confirmed that VEGF expression is upregulated in synovial macrophages and fibroblasts of RA patients, and VEGF expression is positively correlated with RA disease activity and joint destruction (Kanbe et al., 2015). The articular cavity of RA is anoxic microenvironment. Recent studies have shown that the increased expression of HIF-1 in synovium of RA joint is closely related to the occurrence and development of RA (Xu et al., 2010). PI3K-AKT signal pathway is an important intracellular signal transduction pathway, which is closely related to abnormal apoptosis of RA fibroblast-like synovial cells (RAFLS) (Smith and Walker, 2004). Inhibition of abnormally activated PI3K-AKT signaling pathway or expression of anti-apoptotic molecules can induce apoptosis in RAFLS and have therapeutic effect on RA (Liu and Pope, 2003).

Besides the function analysis, we also analysis and validated the key components in KNMSs of each formula. In DSD, the results suggested that the key component isoliquiritigenin from motif 1 (DSD1) exert effect on treatment of RA possibly through acting on MAPK signaling pathway. Studies have shown that isoliquiritigenin suppresses RANKL-induced osteoclastogenesis and inflammatory bone loss *via* RANK-TRAF6, MAPK, IκBα/NF-κB, and AP-1 signaling pathways (Zhu et al., 2012). In GFD, the results suggested that the key component isorhamnetin from motif 1 (GFD1) treats RA possibly through acting on TNF signaling pathway. Published reports confirmed that isorhamnetin play intervening roles in the development and progression of RA *via* anti-inflammatory and anti-oxidative activities. Previous studies have suggested that isorhamnetin attenuates collagen-induced arthritis *via* modulating the levels of cytokines TNF-α, IL-1β, and IL-6 etc. in the joint tissue homogenate of mice (Wang and Zhong, 2015). In HGWD, the results suggested that the key component quercetin from motif 5 (HGWD5) has therapeutic effect on RA possibly through acting on PI3K-Akt signaling pathway. This also verified by previous studies, which found that the mechanisms responsible for the quercetin-induced apoptosis of FLS from patients with RA are associated with the inhibition of PI3K/AKT pathway activation (Pan et al., 2016). Cellular experiments were applied to prove the reliability of the network pharmacology model through verifying the protective effects of key components in KNMSs of three formulas on the inflammation of mice RAW264.7 cells induced by LPS. In addition, in order to better evaluate the reliability of our proposed network pharmacology model, *in vivo* study will be conducted in our future research.

To summarize, a network pharmacology-based approach was established to extract core components group and decode the mechanisms of different formulas treat the same disease of TCM. Additionally, our proposed KNMS prediction and validation strategy provides methodological reference for optimization of core components group and interpretation of the molecular mechanism in the treatment of complex diseases using TCM.

## Data Availability Statement

The raw data supporting the conclusions of this article will be made available by the authors, without undue reservation, to any qualified researcher.

## Author Contributions

A-PL, D-GG, and LG provided the concept and designed the study. K-XW and YG conducted the analyses. K-XW and D-GG wrote the manuscript. K-XW, YG, CL, YL and B-YZ participated in data analysis. X-MQ, G-HD, and A-PL provided oversight. A-PL, D-GG, and LG contributed to revising and proof-reading the manuscript. All authors contributed to the article and approved the submitted version.

## Funding

This study is financially supported by the Startup fund from Southern Medical University (grant No. G619280010), the Natural Science Foundation Council of China (grant No. 31501080), Hong Kong Baptist University Strategic Development Fund [grant No. SDF13-1209-P01, SDF15-0324-P02(b) and SDF19-0402-P02], the Key Laboratory of Effective Substances Research and Utilization in TCM of Shanxi Province (No. 201705D111008-21), Hong Kong Baptist University Interdisciplinary Research Matching Scheme (grant No. RC/IRCs/17-18/04), the General Research Fund of Hong Kong Research Grants Council (grant No. 12101018, 12100719, 12102518).

## Conflict of Interest

The authors declare that the research was conducted in the absence of any commercial or financial relationships that could be construed as a potential conflict of interest.
