# Supplementary material for: Uncovering the Complexity Mechanism of Different Formulas Treatment for Rheumatoid Arthritis Based on a Novel Network Pharmacology Model
Source: Front Pharmacol. 2020 Jul 10;11:1035. doi: 10.3389/fphar.2020.01035 (PMC7365894; doi:10.3389/fphar.2020.01035)
Supplement: Supplementary file 1 [file DataSheet_1.docx]

The chromatograms of herb.

*Angelica sinensis* (Danggui)


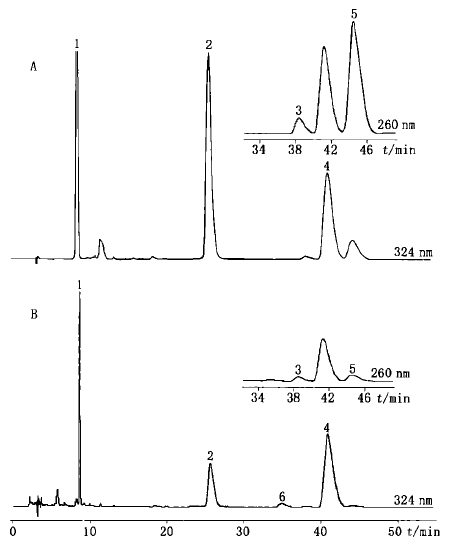


Figure 1 HPLC chromatograms of mix standards (A) and sample (B). 1. Ferulic acid 2. Coniferyl ferulate 3. E-3-butylidenephthalide 4. Z-ligustilide 5. Z-3-butylidenephthalide 6. E-ligustilide.

*Cinnamomi ramulus* (Guizhi)


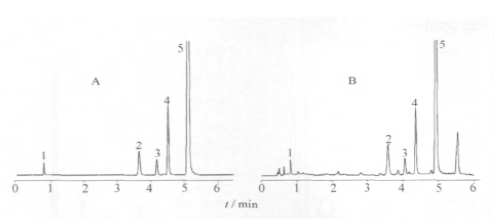


Figure 2 UPLC chromatograms of mix standards (A) and sample (B). 1. Protocatechuic acid 2. Coumarin 3. Cinnamic alcohol 4. Cinnamic acid 5. Cinnamaldehyde.

*Paeonia albiflora* (Baishao)


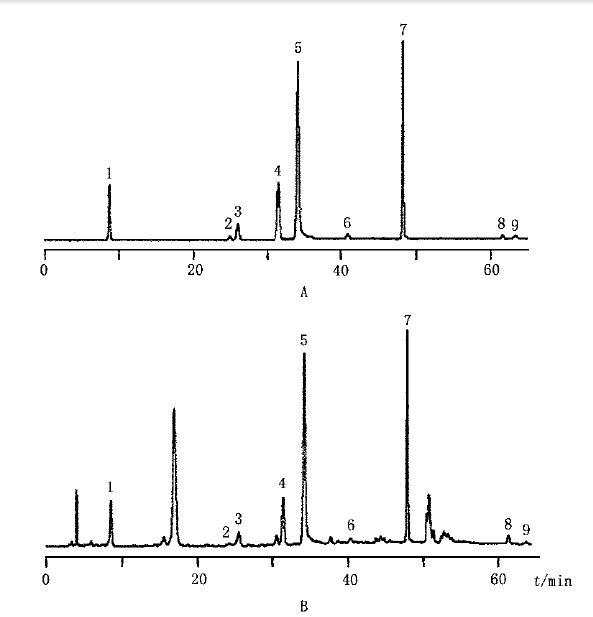


Figure 3 HPLC chromatograms of mix standards (A) and sample (B). 1. Gallic acid 2. Hydroxyl-paeoniflorin 3. Catechin 4. Albiflorin 5. Paeoniflorin 6. Benzoic acid 7 1, 2, 3, 4, 6 -pentagalloylglucose 8. Benzoyl -paeoniflorin 9. Paeonol.

*Asarum sieboldii* (Xixin)


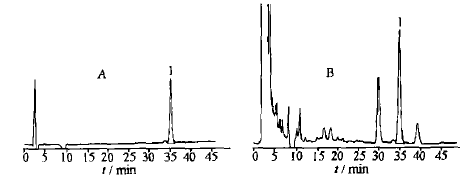


Figure 4 HPLC chromatograms of standards (A) and sample (B). 1. Aristolochic acid A.

*Glycyrrhiza uralensis* (Gancao)


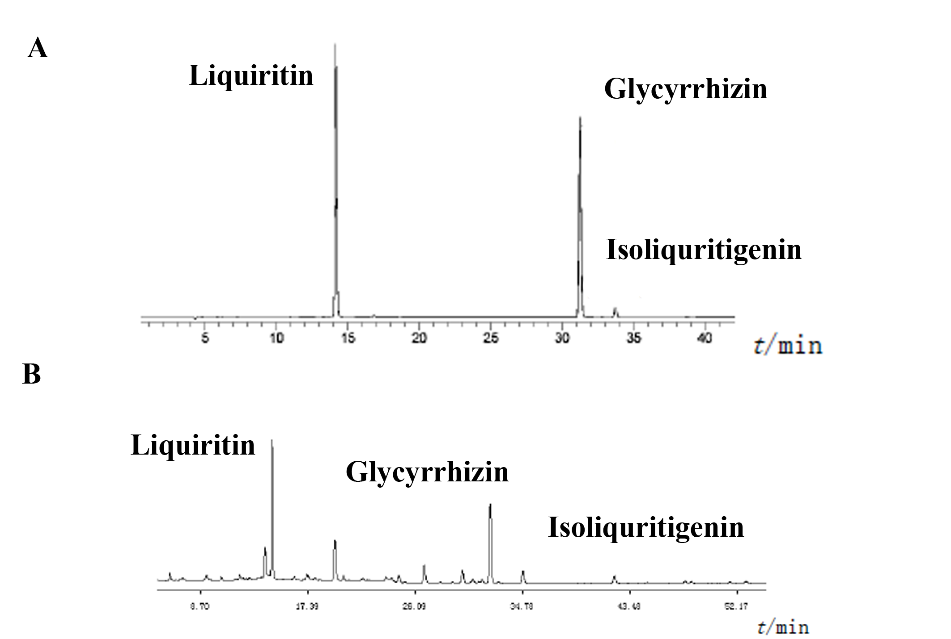


Figure 5 HPLC chromatograms of mix standards (A) and sample (B).

*Medulla tetrapanacis* (Tongcao)


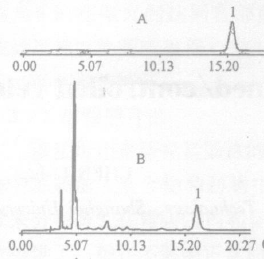


Figure 6 RP-HPLC chromatograms of standards (A) and sample (B). 1. Calceolar ioside B.

*Jujubae fructus* (Dazao)


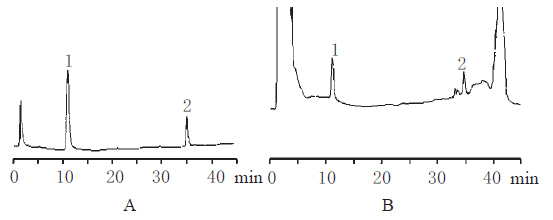


Figure 7 HPLC chromatograms of mix standards (A) and sample (B). 1. Rutin 2. Quercetin 3. Isorhamnetin.

*Aconiti lateralis radix praeparata* (Fuzi)


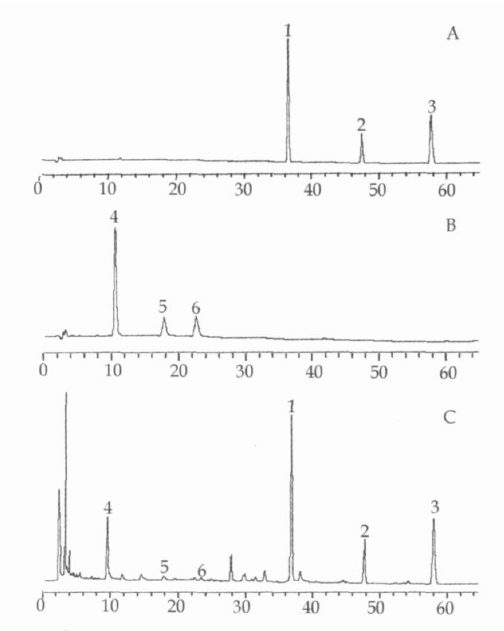


Figure 8 HPLC chromatograms of mix standards (A, B) and sample (C). 1. mesaconitine 2. aconitine 3. hypaconitine 4. benzoylm esaconin 5. Benzoylaconine 6. Benzoylhy paconine.

*Zingiber officinale roscoe* (Shengjiang)


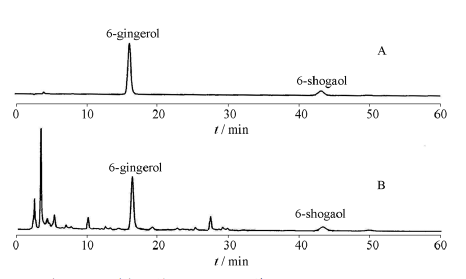


Figure 9 HPLC chromatograms of mix standards (A) and sample (B).

*Astragalus membranaceus* (Huangqi)


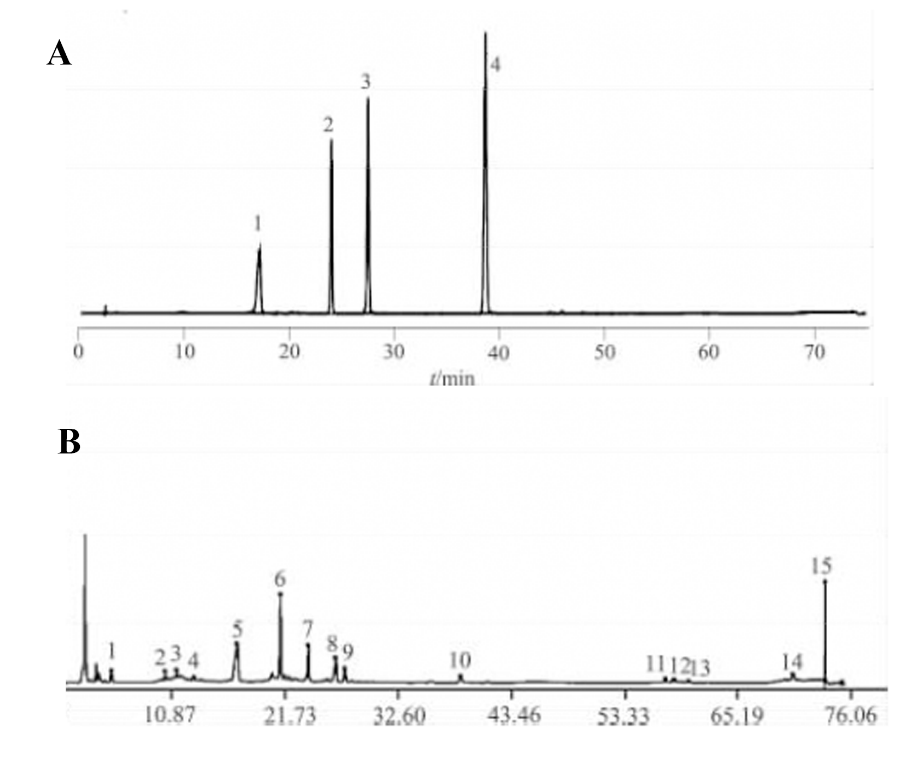


Figure 10 HPLC chromatograms of mix standards (A) and sample (B). 1. Campanulin 4. Formononetin.
