# Supplementary material for: Uncovering the Complexity Mechanism of Different Formulas Treatment for Rheumatoid Arthritis Based on a Novel Network Pharmacology Model
Source: Front Pharmacol. 2020 Jul 10;11:1035. doi: 10.3389/fphar.2020.01035 (PMC7365894; doi:10.3389/fphar.2020.01035)
Supplement: Supplementary file 2 [file Table_1.docx]

Table S1 The detail information of network KNMSs in DSD, GFD and HGWD

| Formula | KNMS | Gene list | P value | FDR value |
| --- | --- | --- | --- | --- |
| DSD | 1 | MAPT,ABCG2,ESR2,TDP1,CBR1,CP1B1,DCOR,CYP19A1,ALOX5,DYRK1A,ALDH2,IL2,ALOX15,XDH,CDK6,CAH4,CAH7,PTGS1,ALOX12,NQO1,T2R31,PTGS2,BACE1,ALOX15B,3MG,CP19A,AOFB,LOX12,NRAM2,PERF,MMP8,GP183,Q16198,KLK14,HPPD,PGFRA,AL1A1,PE2R3,PA21B,MDHC,DYRK3,GRIK3,NAC1,DOPO,ALOXE3,PPAC,PLGF,DHB3,DAPK1,LGUL,NOS1,MDR1,ERR2,TF65,NOS3,VEGFA,HSD17B1,ALOX12B,MIF,MRP1,NEK6,CATL2,NOS2,CAH3,NOX4,HSD11B2,TTHY,AOFA,LOX15,RDH8,HSD17B2,MAOA,ALDR,PDIA1,DHPR,AKR1B1,TNKS1,TBB1,A4,ERN1,BACE2,CAH12,MAOB,AKR1B10,SIAT1,LOX5,CA2,CAH6,TNKS2,CAH13,ALDH1A2,6PGD,CA1,CAH5A,G6PD,CYP1A2,AKR1B15,ULA1,PPBI,NF2L2,CAH14,CBR3,PDE5A,ALDH1L1,ALDH1A1,CRYZ,CYP1B1,AMY1,NUAK1,CP1A1,NQO2,ALDH1B1,FUT4,FUT7,SIAT6,CELA1,TBB3,ABCC2,CYP1A1PDE11A,CAH9,ABCC1,CA9,MOT4,GPR35,TNR1A,CISD1,SCARB1,CA3,AKR1A1,AKR1E2,CP1A2,CAH5B,KDM4E,KS6A3,ERR1,ALDH1A3,ACHA7,CA13,PCSK7,ABCC3,MTR1A,BGLR,SSDH,GABT,LPA,CA14,CA5A,CA5B,CA7,CA12,NEMO,IMDH1,IMDH2,F263,ABCC9,FACE2,ALDH1L2,AOX1,TPMT,CA4,PLA2G1B,KCC2B,GFER,C11B1,DHB1,I23O1,PLAP,CHK1,CTNB1,CP3A4,TSSK2,TBXAS1,PPBT,CD5R1,SYUA,PA2G5,KCJ11,C11B2,GBRA3,GBRB3,GBRG2,S22AC,CLTR1,KAT3,TAU,CA6,HS71A,MPO,DHB2,P85A,UFO,CSK21,FAK1,VGFR2,CP27A,NPBW1,PA2GA,CTDS1 | 0.0001 | 0.000475 |
| DSD | 2 | ESR1,SRC,EP300,JUN,STAT3,HDAC1,AKT1,XPO1,RELA,AR,SMAD3,HDAC3,PRKCA,YWHAG,MDM2,STAT1,MAPK14,GSK3B,PRKDC,NFKB1,MAPK8,PRKCD,HDAC2,CREB1,NR3C1,CDK1,TOP2A,JUNB,NCOA1,HIF1A,JUND,PRKCB,RAD52,CDC25A,RAF1,PCNA,HDAC5,KAT2A,HDAC4,MAPK9,CASP3,HDAC6,CDC25B,RARA,MAPK10,TOP1,PRKCQ,FOXO1,MAPK11,NR0B2,TOP2B,HNF4A,PKN1,GSK3A,HDAC7,PRKCG,RPS6KA5,PDE4D,HDAC9,PPARG,REL,STAT6,NEK2,CDK8,CSNK1D,APEX1,FEN1,NR0B1,CDC25C,MAPKAPK2,TERT,ERCC5,RPS6KA3,NR4A2,KLF5,LDHA,AKT3,ESRRA,SENP1,PGR,HDAC8,PPARA,APLP2,BCL2,VDR,GLI2,RPS6KA2,EPAS1,PPARD,TNF,MCL1,SENP7,AHR,SREBF1,CSNK1A1,MALT1,MAPKAPK3,ESRRG,BCL2L1,CDK3,NR1H4,NR1H3,SREBF2,TRPC6,PDE4C,AURKB,THRB,EIF4A1,RARB,PRKCH,POLB,NR1H2,RARG,SENP6,TRPC3,PIM1,RPS6KA4,EIF4A2,MAPKAPK5,ESRRB,ACP1,NLRP1,RORA,MAP2,EIF4A3,TAOK1,NR3C2,ALK,PAX8,KDM4A,CASP7,PRTN3,MME,GLI1,C1R,FNTA&FNTB | 0.0001 | 0.00095 |
| DSD | 3 | AK1C3,TRPM2,PYGL,KCC2A,TOPK,DYRK4,PACR,PE2R2,HDA11,TRPV1,ABCC8,AK1C4,PGH2,DYN1,PD2R,ALR,HPSE,MPRI,PE2R1,G6PT1,HDA10,PTN7,DUS3,B2LA1,GLR,LT4R1,BKRB2,PGH1 | 0.004763 | 0.012928 |
| DSD | 4 | LEG9,CD22,TYRO,S28A3,SLC5A1,SLC5A2,LEG7,SLC5A11,SLC5A4,SLC5A10,SLC5A3,SLC5A9,SC5A2,SC5A1,LEG3,RASN,SC5A4,SC5AB,B4GT1,IF4A1,LEG4,LEG8,TYDP1,AMYP,SLC28A3,SLC28A1,SLC28A2,TYR,RASH | 0.0001 | 0.000475 |
| DSD | 5 | SHBG,DHCR7,MMP26,SIGMAR1,CYP51A1,FDFT1,PTCA,KCNN1,KCNN2,CYP2J2,S22A3,MTR1B,RBBP9,OX2R,OX1R,TSPO,MMP15,MMP16,KLK5,TY3H,FKB1A,ACHA2,VMAT2,5HT1A,5HT7R,KCNN3,MTLR,TA2R,CHRNB2&CHRNA4,5HT2A,ACKR3,NPM,5HT1B,DCLK2,5HT2C,MK07,5HT1D | 0.000621 | 0.001966 |
| DSD | 6 | HMGCR,CYP17A1,CAH1,CAH2,LRP8,SHH,HSD17B3,CP24A,EBP,LDLR,FADS1,VLDLR,SRD5A2,CP27B,S5A1,S5A2,ANDR,PUR9,ABCBB,CDC45,SIA4A,DPOLA,NTCP2,MPIP1,NPC1,NPCL1,ORG,SRBP2,VTDB | 0.011595 | 0.027537 |
| DSD | 7 | NMUR2,ADCY5,ADCY1,FPR2,ADORA2A,BDKRB1,GRM6,ADORA1,ADORA2B,DRD2,OPRD1,DRD3,OPRM1,DRD4,CCR4,OPRK1,OPRL1,CHRM2,CHRM4,ADRA2A,MTNR1A,MTNR1B,CXCR1,ADORA3,CNR1,HTR1A,HTR1B,HRH3,GPR55,CNR2,ADRA2B,ADRA2C,PTAFR,ADRA1B,ADRA1A,ADRA1D,CHRM5,CHRM3,EDNRB,EDNRA,F2,CHRM1,HTR2A,LTB4R,LTB4R2,HTR2C,MLNR,GHSR,FFAR1,ADRB2,DRD1,ADRB1,DRD5,PTGER2,PTGIR,HTR7,ADRB3,HRH2,PDE4A,TAAR1,GPBAR1,PDE4B,APP,PDE1A | 0.0001 | 0.000633 |
| GFD | 1 | MAPT,ABCG2,TDP1,ESR2,CBR1,CP1B1,DCOR,CYP19A1,ALOX5,DYRK1A,XDH,ALDH2,CAH7,IL2,CAH4,ALOX15,PTGS1,NQO1,T2R31,ALOX12,3MG,ALOX15B,LOX12,PTGS2,CDK6,CP19A,PPAC,LGUL,ALOXE3,DHB3,PLGF,ERR2,TF65,MDR1,MIF,DAPK1,NOX4,MRP1,NEK6,LOX15,HSD17B1,CATL2,ALOX12B,TTHY,CAH3,VEGFA,AOFA,RDH8,DHPR,ALDR,PDIA1,LOX5,HSD11B2,AKR1B1,TNKS1,HSD17B2,MAOA,AKR1B10,CAH13,SIAT1,BACE2,TNKS2,ALDH1A2,MAOB,CAH12,AKR1B15,ULA1,PPBI,CBR3,ALDH1L1,ALDH1A1,CYP1A2,AMY1,ALDH1B1,6PGD,MALT1,MOT4,NUAK1,CELA1,TBB3,G6PD,PDE11A,SCARB1,ABCC2,CYP1B1,GPR35,ABCC1,DE5A,FUT4,SIAT6,FUT7,AKR1A1,AKR1E2,KDM4E,KS6A3,NU4M,ALDH1A3,ERR1,CYP1A1,PCSK7,MTR1A,BGLR,ABCC3,IMDH1,IMDH2,F263,GFER,ABCC9,ALDH1L2,AOX1,KCC2A,TOPK,DYRK4,PLA2G1B,KCC2B,DHB1,I23O1,PLAP,CHK1,CTNB1,CAH2,TPMT,CD5R1,KCJ11,GBRA3,GBRB3,GBRG2,S22AC,KAT3,TSSK2,PA2G5,SAE1&UBA2,HS71A,MPO,DHB2,P85A,UFO,CSK21,FAK1,VGFR2,MMP8,PA21B,MDHC,PPBT,NAC1,DOPO | 0.0001 | 0.000367 |
| GFD | 2 | NMUR2,ADCY5,ADCY1,ADORA2A,FPR2,GRM6,LPAR3,LPAR1,LPAR2,ADORA2B,ADORA1,DRD2,OPRD1,DRD3,DRD4,CHRM2,CCR4,OPRM1,CHRM4,OPRK1,OPRL1,ADRA2A,ADORA3,HTR1A,HTR1B,MTNR1A,MTNR1B,CNR2,CXCR1,HRH3,GPR18,CNR1,GPR55,ADRA2B,ADRA2C,S1PR2,S1PR4,OXER1,GPR50,PDE1A,ADRA1B,ADRA1A,ADRA1D,CHRM5,CHRM3,CHRM1,EDNRB,EDNRA,HTR2A,F2,PTAFR,LTB4R2,HTR2C,MLNR,GHSR,FFAR1,LTB4R,ADRB2,DRD1,ADRB1,DRD5,FSHR,PTGER2,PTGIR,HTR7,ADRB3,TAAR1,HRH2,GPBAR1,KCNK2,ADCYA | 0.0001 | 0.000314 |
| GFD | 3 | AOFB,BACE1,AK1BA,TBB1,A4,NF2L2,CAH6,TF,CP1A1,CAH5A,CAH14,TLR9,ERN1,CISD1,CAH5B,CRYZ,TNR1A,NQO2,CP1A2,CAH9,ACHA7,NEMO,NLRP1,SSDH,GABT,TRPM2,PE2R4,AK1C3,PYGL,EST2,PACR,PE2R2,HDA11,ABCC8,AK1C4,PGH2,SYUA,CLTR1,TAU,DYN1,PD2R,ALR,HPSE,MPRI,PE2R1,G6PT1,HDA10,B2LA1,GLR,BKRB2,PGH1,GRIK3,GP183,Q16198,KLK14,HPPD,PGFRA,AL1A1,PE2R3 | 0.003231 | 0.007107 |
| GFD | 4 | SRC,EGFR,PTPN1,STAT3,PTPN2,FYN,ERBB2,PTPN11,STAT1,ERBB4,ERBB3,FLT1,PDPK1,PLCG1,KDR,PRKCD,YES1,AKT2,MET,FLT4,RAF1,FGFR2,PLCG2,FGFR1,PTPN6,FGFR3,ODC1,FGR,KIT,PRKCQ,FGFR4,AZIN1,FGF2,IGF1R,FGF1,HCK,ADC,MAPKAPK2,CSF1R,NDUA4,PRKCH,CTSK,FRK,CTSL1,CTSS,LX15B,CTSL2,ALK,MAPKAPK3,MAPKAPK5,ADA2C,MAP2,ADA2A,FLT3,HS90A | 0.0001 | 0.00044 |
| GFD | 5 | SLC6A2,SLC6A3,MMP26,DPP4,PTCA,MTR1B,RBBP9,OX2R,OX1R,KCNN1,KCNN2,FAP,MTLR,MMP15,TA2R,TY3H,FKB1A,5HT1A,5HT7R,ACHA2,VMAT2,MMP16,CHRNB2&CHRNA4,5HT2A,ACKR3,NPM,5HT1B,DCLK2,5HT2C,KCNN3,MK07,5HT1D | 0.0001 | 0.0011 |
| GFD | 6 | SHBG,S22A3,1433G,TRPV1,KLK5,PTN7,DUS3,LT4R1,LYPA2,LYPA1,PA24B,THB,THA,GP174,P2Y10,UBCP1,GPR34,FAAH1,MPEG1,PA24C,EBPL,FABPH,HAOX1 | 0.001217 | 0.002975 |
| GFD | 7 | PDE4A,PDE4B,PDE4D,KCNA3,KCNA4,NEK2,KCNA5,KCNA2,KCNA1,PDE4C,KCNH2,KCNH6,KCNH7,SCN3A, KCNA10,KCNA6,KCNA7 | 0.003772 | 0.007545 |
| GFD | 8 | CD22,TYRO,LEG9,S28A3,SLC5A2,SLC5A11,SLC5A4,SLC5A10,SLC5A3,SLC5A9,SC5A2,SC5A1,LEG3,RASN,SC5A4,SC5AB,B4GT1,IF4A1,LEG4,LEG8,LEG7,TYDP1,AMYP,SLC28A3,SLC28A1,SLC28A2,TYR,RASH | 0.0001 | 0.000733 |
| GFD | 9 | SLC5A1, MGAM, SI, CHRNA7, GBA, GAA, CHRFAM7A, GBA2, UGCG, FUCA1, GLB1, FUCA2, GLB1L, MAN2B1 | 0.000233 | 0.000642 |
| GFD | 10 | SLC6A9, CHLE, ANO1, SLC6A7, SLC6A5, SLC6A14, UBP4, PTN9, UBP5, PTN6, CC14A, PTN22, MP2K1, PTN13, FABP5 | 0.013375 | 0.024521 |
| HGWD | 1 | HSP90AA1,ESR1,HSP90AB1,SRC,EP300,STAT3,JUN,EGFR,HDAC1,AR,RELA,PRKCA,SMAD3,STAT1,AKT1,FYN,NR3C1,ERBB2,GSK3B,HDAC2,NFKB1,HDAC3,CDK1,TDP1,HDAC6,JUNB,ESR2,JUND,RAD52,HDAC5,NCOA1,TOP2A,PRKCB,PRKCD,PTPN1,ERBB4,HDAC4,YES1,KAT2A,ERBB3,CREB1,KDR,STAT2,FOXO1,MET,HDAC7,FLT1,CDC25A,PTPN2,RPS6KA5,TOP2B,CDC25B,PRKCQ,NR0B1,HDAC9,HSP90B1,VDR,PDE4D,STAT6,NFKB2,RPS6KA3,NEK2,PGR,SREBF1,HNF4A,FLT4,RPS6KA2,PKN1,AHR,GLI2,KLF5,IGF1R,APEX1,FGR,VEGFA,DYRK1A,REL,EIF4A1,SENP1,NEK6,CDC25C,CD4,KIT,NR1H4,PDE4C,HDAC8,FRK,CSF1R,EIF4A2,NR3C2,RORA,NR1H3,SREBF2,PIM1,AURKB,THRB,TERT,POLH,RARB,EIF4A3,NR1H2,CDK6,NR4A2,ALK,SENP7,PAX8,RPS6KA4,LDLR,POLB,GLI1,MAP2,SPHK1,LRP8,TLR9,DYRK4,DCLK2,NLRP3,POLK,NQO2 | 0.0001 | 0.0003 |
| HGWD | 2 | G6PD,CAH1,CAH2,GPBAR,ALDH2,CP24A,EBP,CP19A,CBG,NTCP,VLDLR,EBPL,CP27B,S5A1,S5A2,ANDR,PUR9,ABCBB,CDC45,SIA4A,DPOLA,NTCP2,MPIP1,MRP4,NPC1,NPCL1,RORG,SRBP2,VTDB,ATP1A3,ATP4A,ATP1A2,ATP1A4,AT12A,SO4C1,AT1A1 | 0.0001 | 0.000525 |
| HGWD | 3 | DHPR,CP1B1,BACE1,CA2,CBR1,CA1,T2R31,1433G,CAH5A,PLGF,CAH7,FUT7,BACE2,CAH12,CAH3,CAH9,6PGD,FUT4,CA13,CA14,SIAT6,CA9,PPBT,CA4,CA5A,CA5B,CA7,CA12,SIAT1,CA6,CP3A4,MMP27,MMP20 | 0.000149 | 0.000392 |
| HGWD | 4 | ADCY5,ADCY1,FPR2,NMUR2,GRM6,LPAR3,LPAR1,LPAR2,ADORA2A,DRD2,OPRD1,DRD3,OPRM1,DRD4,ADORA1,OPRK1,OPRL1,CHRM2,ADRA2A,CNR1,CHRM4,HTR1A,HTR1B,MTNR1A,MTNR1B,CNR2,CXCR1,CCR4,HRH3,GPR18,ADORA3,ADRA2B,ADRA2C,S1PR2,S1PR4,OXER1,F2,PTAFR,ADRA1B,ADRA1A,CHRM5,ADRA1D,CHRM3,CHRM1,EDNRA,HTR2A,HTR2C,MLNR,GHSR,EDNRB,PDE3A,PDE1A,PDE1B | 0.0001 | 0.00035 |
| HGWD | 5 | DAPK1,CAH4,MIF,TTHY,TNKS2,CA3,AOFA,ERR2,ALDR,NUAK1,MRP1,PPAC,3MG,ULA1,TNKS1,AMY1,PPBI,GPR35,PLA2G1B,FLT3,IL2,MOT4,PCSK7,CD5R1,HS71A,MPO,DHB2,P85A,UFO,KCC2B,CSK21,FAK1,VGFR2,PDIA1,SYUA,KDM4E,KS6A3,F263 | 0.0001 | 0.0003 |
| HGWD | 6 | PRKCG,SLC6A2,SLC6A3,ABCB1,SLC6A4,ABCB5,ABCB11,ABCB4,SLC6A9,BCHE,SLC6A7,LEG1,GRP3,SLC6A5,SLC6A14,ACHE,LEG9,KPCA,RYR1,MA2B1,LEG7,LGALS9,LGALS9B,LGALS9C,ACHA5 | 0.003425 | 0.006539 |
| HGWD | 7 | TF,MDR1,PTCA,MMP26,MAOB,OX2R,OX1R,KCNN1,KCNN2,MAOA,RBBP9,MMP15,TY3H,FKB1A,5HT1A,5HT7R,ACHA2,VMAT2,MTR1B,TSPO,MMP16,MTLR,TA2R,CHRNB2&CHRNA4,5HT2A,ACKR3,NPM,5HT1B,ADA2A,5HT2C,KCNN3,MK07,5HT1D | 0.0001 | 0.00105 |
| HGWD | 8 | CAH6,LGUL,TRPV1,CISD1,LOX15,NF2L2,DHB3,A4,NEMO,PTN7,DUS3,LT4R1,LOX5,LYPA2,LYPA1,PA24B,THB,THA,RDH8,GP174,P2Y10,UBCP1,GPR34,FAAH1,MPEG1,PA24C,FABPH,HAOX1 | 0.0001 | 0.0007 |
| HGWD | 9 | CAH14,CAH5B,PYGL,AOFB,CP1A1,PACR,TNR1A,S28A3,PE2R2,AK1C3,DYN1,HDA11,PD2R,ALR,HPSE,IMDH1,PE2R4,MPRI,IMDH2,AK1C4,PE2R1,G6PT1,CAH13,HDA10,KCC2A,B2LA1,GLR,TF65,TOPK,BKRB2,CHLE,PGH2,PGH1 | 0.021002 | 0.036753 |
| HGWD | 10 | ABCG2,AKR1B1,XDH,NOX4,SHH,ABCC1,AKR1B10,AKR1B15,AKR1A1,ABCC2,CYP1A2,AKR1E2,TGS1,ABCC8,AOX1,ABCC3,SAE1&UBA2 | 0.00086 | 0.002008 |
