# Supplementary material for: Uncovering the Complexity Mechanism of Different Formulas Treatment for Rheumatoid Arthritis Based on a Novel Network Pharmacology Model
Source: Front Pharmacol. 2020 Jul 10;11:1035. doi: 10.3389/fphar.2020.01035 (PMC7365894; doi:10.3389/fphar.2020.01035)
Supplement: Supplementary file 4 [file Table_3.docx]

**Table S3** The value of network KNMSs in DSD, GFD and HGWD.

| **Formulas** | KNMS | **d_c_** | **R** | **CS** |
| --- | --- | --- | --- | --- |
| DSD | 1 | 2322 | 14.34 | 66.34% |
| DSD | 2 | 60 | 0.11 | 0.53% |
| DSD | 3 | 117 | 1.00 | 4.63% |
| DSD | 4 | 87 | 0.60 | 2.78% |
| DSD | 5 | 127 | 0.87 | 4.01% |
| DSD | 6 | 62 | 0.36 | 1.67% |
| DSD | 7 | 35 | 0.10 | 0.48% |
| GFD | 1 | 1828 | 15.28 | 48.69% |
| GFD | 2 | 57 | 0.47 | 1.48% |
| GFD | 3 | 410 | 3.47 | 11.07% |
| GFD | 4 | 268 | 2.18 | 6.95% |
| GFD | 5 | 145 | 1.22 | 3.87% |
| GFD | 6 | 91 | 0.77 | 2.45% |
| GFD | 7 | 19 | 0.16 | 0.49% |
| GFD | 8 | 87 | 0.73 | 2.34% |
| GFD | 9 | 42 | 0.34 | 1.07% |
| GFD | 10 | 54 | 0.46 | 1.46% |
| HGWD | 1 | 45 | 0.03 | 0.43 % |
| HGWD | 2 | 70 | 0.41 | 5.99 % |
| HGWD | 3 | 103 | 0.46 | 6.70 % |
| HGWD | 4 | 15 | 0.01 | 0.14 % |
| HGWD | 5 | 149 | 1.17 | 17.26 % |
| HGWD | 6 | 109 | 0.24 | 3.57 % |
| HGWD | 7 | 127 | 0.83 | 12.13 % |
| HGWD | 8 | 91 | 0.61 | 8.99 % |
| HGWD | 9 | 117 | 1.00 | 14.69 % |
| HGWD | 10 | 76 | 0.06 | 0.86 % |
